# Molecular evolution of neuropeptides in the genus *Drosophila*

**DOI:** 10.1186/gb-2008-9-8-r131

**Published:** 2008-08-21

**Authors:** Christian Wegener, Anton Gorbashov

**Affiliations:** 1 Emmy Noether Neuropeptide Group, Animal Physiology, Department of Biology, Philipps-University, Karl-von-Frisch-Strasse, D-35032 Marburg, Germany

## Abstract

**Background:**

Neuropeptides comprise the most diverse group of neuronal signaling molecules. They often occur as multiple sequence-related copies within single precursors (the prepropeptides). These multiple sequence-related copies have not arisen by gene duplication, and it is debated whether they are mutually redundant or serve specific functions. The fully sequenced genomes of 12 *Drosophila *species provide a unique opportunity to study the molecular evolution of neuropeptides.

**Results:**

We data-mined the 12 *Drosophila *genomes for homologs of neuropeptide genes identified in *Drosophila melanogaster*. We then predicted peptide precursors and the neuropeptidome, and biochemically identified about half of the predicted peptides by direct mass spectrometric profiling of neuroendocrine tissue in four species covering main phylogenetic lines of *Drosophila*. We found that all species have an identical neuropeptidome and peptide hormone complement. Calculation of amino acid distances showed that ortholog peptide copies are highly sequence-conserved between species, whereas the observed sequence variability between peptide copies within single precursors must have occurred prior to the divergence of the *Drosophila *species.

**Conclusion:**

We provide a first genomic and chemical characterization of fruit fly neuropeptides outside *D. melanogaster*. Our results suggest that neuropeptides including multiple peptide copies are under stabilizing selection, which suggests that multiple peptide copies are functionally important and not dispensable. The last common ancestor of *Drosophila *obviously had a set of neuropeptides and peptide hormones identical to that of modern fruit flies. This is remarkable, since drosophilid flies have adapted to very different environments.

## Abstract 2

The first genomic and chemical characterization of fruit fly neuropeptides outside *Drosophila melanogaster* provides insights into the evolution of the neuropeptidome in this genus.

## Background

Neuropeptides comprise the most diverse group of intercellular signaling molecules in eumetazoan animals and regulate vital physiological processes as hormones, neuromodulators or neurotransmitters. Since neuropeptides are too small to be directly channeled into the regulated secretory pathway, they are post-translationally processed from larger prepropeptides by enzymatic cleavage.

In vertebrates, gene or genome duplications are main events that have led to the diversity of neuropeptides [1-4]. Over time, each prepropeptide gene acquires nucleotide substitutions that - if inside a peptide-coding sequence and not synonymous - will result in altered peptide sequence. If the peptide's function is vital and interference with peptide signaling decreases Darwinian fitness, there will be stabilizing selection on at least that part of the peptide sequence responsible for receptor binding and activation. In consequence, the peptide sequence will be conserved over time [4]. In fact, the sequences of many ortholog neuropeptides, such as oxytocin or somatostatin, have been highly conserved throughout vertebrate phylogeny [4]. However, considerable sequence variation can be found between duplicated peptides of a family, for example, in the growth hormone-releasing factor superfamily [5]. According to a classic model of molecular evolution [6], this is because a duplicated peptide sequence may be able to escape from natural selection and drift neutrally [7] if its original function is maintained by its paralog. In principle, the mutations accumulating in the 'escaped' peptide sequence may then lead to nonfunctionalization, subfunctionalization or neofunctionalization by acquisition of new features such as altered half-life, altered receptor binding kinetics, altered tissue expression patterns (for example, neuropeptides of the NPY family or the POMC prepropeptide [1,8]) or receptor specificities by peptide-receptor co-evolution [9,10]. If sub- or neofunctionalized, the new peptide will undergo positive selection for the new function and so become constrained by purifying selection. If the increased amount of peptides resulting from the duplication is beneficial, the duplicated peptide may also immediately increase Darwinian fitness prior to an accumulation of sequence mutations ('more-of-the-same') [10,11].

A special feature of many neuropeptides that cannot be explained by gene duplication is the occurrence of multiple members of one peptide family within a single prepropeptide. For example, vertebrate prepropeptides encoding, melanocortins, hypocretins, RFamides or tachykinins, contain two to a few members of a single peptide family [12]. In invertebrates, copy numbers can reach even higher numbers. Examples include 37 related peptides from the metamorphosin A precursor of the sea anemone *Anthopleura elegantissima *[13], 24 different FMRFa-like peptides encoded by the *fmrf *gene of the cockroach *Periplaneta americana *[14], 35 FGLamides from the allatostatin precursor of the prawn *Macrobrachium rosenbergii *[15], up to nine RFamides encoded per *flp *genes of *Caenorhabditis elegans *[16], and 35 enterins contained in the enterin precursor of *Aplysia *[17]. These multiple copies are encoded on the same gene, and often even on the same exon. They most likely have arisen by unequal recombination between nearly identical nucleotide stretches. This has the important consequence that, unlike peptides generated by gene or genome duplication, these copies cannot move to a new genomic location and acquire promoter-driven differential spatial or temporal expression patterns since they are encoded on the same gene, and they cannot be specifically silenced when located on the same exon. Multiple copies are thus equal at birth, at least on the genetic level [18]. Unlike for peptides originating from whole genome duplications, there is also no co-duplicated receptor as a directly available partner for sub- or neofunctionalization. It is therefore difficult to fit them directly into the established models of molecular evolution for duplicated peptide genes [1,2,4].

At least two questions arise from this: is the molecular evolution of multiple copy neuropeptides similar to that of duplicated peptides? And more importantly, what is the functional significance of the individual multiple copies contained in given prepropeptides - a long-standing problem in invertebrate neuroendocrinology (see, for example, [19-22]). At one extreme, each peptide copy may have its unique and specific function, receptor or expression pattern. On the other extreme, peptide copies may be functionally redundant if they are co-expressed, co-released and also share an identical effect space [21]. Among others, studies on the effect of multiple co-expressed peptide copies on the neuromuscular junction of *Aplysia *and *Drosophila *provide evidence for such a redundancy [22,23], but differential activities might be found when looking at, for example, different developmental times or target sites. In fact, other studies speak against a functional redundancy, and report differential target-specific effects of multiple copy peptides in insects and molluscs (for example, [19,24-26]).

**Figure 1:**
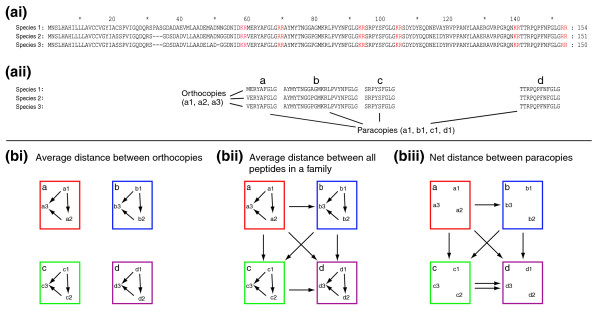
Terminology and amino acid distances. **(ai) **Peptide copy terminology exemplified by three aligned ASTa prepropeptides from species a1-3. **(aii) **Processing at dibasic processing sites (indicated in red in (ai)) yields the four neuropeptides ASTa1-4. The C-terminal glycine is further processed to yield the C-terminal amidation. Peptide copies aligning at the same position in the precursor (for example, ASTa1 of species a1-3) will be referred to as orthocopies, which do not have to be sequence-identical. The different copies in a precursor of a single species are paracopies (for example, ASTa1-4 of species a1) = not at the same location. Paracopies may or may not be sequence-identical. **(b) **Different types of amino acid distances obtained by pairwise comparisons. **(bi) **The average distance D_o _between orthocopies is the arithmetic mean of all individual pairwise distances. It does not contain distances between different paracopies. **(bii) **The average distance between all peptides within a family D_f _is the arithmetic mean of all individual pairwise distances. It contains all pairwise distances between orthocopies and all paracopies. **(biii) **The net distance D_np _between paracopies is similar to D_f _after subtraction of D_o_. It does not contain the pairwise distances between each set of orthocopies.

To comprehensively investigate whether multiple peptide copies are functionally redundant is extremely difficult by experimental means, especially since peptide copies can show different half-lives in the circulation after release (for example, [27]), or differentially activate the same receptor (for example, [28]). It is also difficult to assess the functional importance of individual copies by genetic means since common techniques target the whole gene. We here have chosen an evolutionary and comparative genomic approach to address the functional significance of multiple peptide copies. This opportunity has recently become possible with the publication of the genomes of 12 different *Drosophila *species [29]. A standard nomenclature that refers to multiple peptides belonging to the same peptide family located on the same precursor does not exist. Based on [30], we will use the following terminology (see Figure 1): peptide copies aligning at the same position within the precursors of different species will be referred to as orthocopies. Orthocopies do not have to be sequence identical. The different peptide copies within a prepropeptide of a single species are paracopies (that is, not at the same location). The term 'isoform', which has often been used in conjunction with insect neuropeptides, will be avoided because of its differing usage in protein nomenclature.

We mined the *Drosophila *genome database [31] for genes encoding homologs of all known *D. melanogaster *neuropeptide precursor (prepropeptides) encoding neuropeptides up to a size of 50 amino acids. The investigated species belong to the *Drosophila *and *Sophophora *subgenera that diverged 40-60 million years ago [32,33] and contain 97% of the more than 1,000 *Drosophila *species [34]. We then predicted ortho- and paracopies and analyzed their amino acid sequence variation. This is appropriate since most selection pressure is on the peptide sequence and not on the underlying DNA sequence with its often redundant third codon position. Our reasoning was as follows: if peptides are functionally important and their loss decreases Darwinian fitness, their sequence will be under stabilizing selection and hence their sequence will be conserved in the different species. If peptides have no functional importance and their (functional) loss does not affect fitness, they will be able to escape selection pressure and will accumulate sequence variations during *Drosophila *radiation. Thus, if peptide copies are functionally unimportant, we expect a high sequence variation between at least some orthocopies that were able to escape from selection pressure since one or several of their fellow paracopies 'do the job' and hence are under stabilizing selection. This in consequence would lead to an increased sequence variation between paracopies. If peptide copies have a functional importance, we expect low sequence variation between all orthocopies due to stabilizing selection. If the different paracopies activate different receptors or induce different receptor conformations that lead to activation of different intracellular signaling pathways, we expect at the same time an increased sequence variation between paracopies due to subfunctionalization. If peptide copies are individually redundant but functionally important along the 'more-of-the-same' concept, we expect low sequence variation between both ortho- and paracopies.

Our study assumes that neuropeptides are expressed and processed as predicted *in silico *from the genome. This is not given *per se*, since neuropeptides can undergo differential splicing and post-translational processing. To biochemically underpin our assumption in a manageable amount of time, direct MALDI-TOF (matrix-assisted laser desorption ionization-time of flight) mass spectrometric peptide profiling lends itself as a fast and reliable method. We therefore directly profiled the major neuropeptide release sites of four species covering the main *Drosophila *lineages. In *D. melanogaster*, these sites contain about 50% of all biochemically identified neuropeptides and the majority of peptide hormones [35-37].

Our data provide a first genomic prediction of neuropeptides and prepropeptides, and the first chemical neuropeptide characterizations for the newly sequenced *Drosophila *species.

The results suggest that both the peptidome and the peptide hormone complement are conserved throughout *Drosophila*, and that the degree of sequence variation corresponds well with the pharmacological efficacy of the peptides. This provides molecular evidence for a general functional importance of multiple paracopies.

## Results

### Genomics and peptide prediction

We mined the genomes of the 11 newly sequenced *Drosophila *species for homologs of the *D. melanogaster *peptide precursor genes *Akh *(CG1171), *Ast *(CG13633), *Ast-C *(CG149199), *capa *(CG15520), *Ccap *(CG4910), *Crz *(CG3302), *Dh *(CG8348), *Dh31 *(CG13094),*ETH *(CG18105), *Fmrf *(CG2346), *hug *(CG6371), *IFa *(CG33527), *Leucokinin *(CG13480), *Mip *(CG6456), *Dms *(CG6440), *npf *(CG10342), *Nplp1 *(CG3441), *Pdf *(CG6496), *Proct *(CG7105), *Dsk *(CG18090), *sNPF *(CG13968), and *Dtk *(CG14734). We then predicted the encoded neuropeptides; an overview of their numbers is given in Table 1. With the exception of the FMRFa-like peptides (see below), the analyzed genes code for the same number of neuropeptides in each species (43 in total, plus 10-17 FMRFa-like peptides). The translated coding sequences for the prepropeptides and predicted peptides are given as Additional data files 1 and 2.

**Table 1:** Peptide genes and encoded peptides

Prepropeptide gene	Encoded peptide families (number of paracopies)	Paracopies (length)	Amidation signal
*Adipokinetic hormone (AKH)*	AKH (1)	AKH (8)	Y
*Allatostatin A (ASTa)*	ASTa (4)	ASTa-1 (8)	Y
		ASTa-2 (21)	Y
		ASTa-3 (8)	Y
		ASTa-4 (11)	Y
*Allatostatin C (ASTc)*	ASTc (1)	ASTc (15)	N
*Capability (CAPA)*	Periviscerokinins- PVKs (2)	CAPA-PVK-1 (12)	Y
		CAPA-PVK-2 (9-10)	Y
	Pyrokinins - PKs (1)	CAPA-PK (15)	Y
*Crustacean cardioactive peptide (CCAP)*	CCAP (1)	CCAP (9)	Y
*Corazonin*	Corazonin (1)	Corazonin (11)	Y
*Diuretic hormone*_*31*_ *(DH*_*31*_*)*	Diuretic hormones (1)	DH_31 _(31)	Y
*Diuretic hormone*_*44*_	CRF-related hormones (1)	DH_44 _(44)	Y
*Drosokinin*	Kinins (1)	Drosokinin (15)	Y
*Ecdysis-triggering hormone (ETH)*	ETHs (2)	ETH-1 (17-18)	Y
		ETH-2 (12-15)	Y
*Fmrf*	FMRFa-like peptides (10-17)*	(6-11)	Y
*Hugin*	pyrokinins (1)	HUG-PK (8)	Y
*IFamide*	IFamides (1)	IFamide (12)	Y
*Myoinhibiting peptide (MIP)*	MIPs (5)	MIP-1 (9)	Y
		MIP-2 (9)	Y
		MIP-3 (13)	Y
		MIP-4 (11)	Y
		MIP-5 (10)	Y
*Myosuppressin (MS)*	MS (1)	MS (10)	Y
*Neuropeptide F (NPF)*	NPF (1)	NPF (36)	Y
*NPLP1*	'ASP' (1)	'ASP' (13-15)	N
	PNamides (1)	PNamide (13-15)	Y
	'MTYamides' (1)	'MTYamide' (14)	Y
*Pigment-dispersing factor (PDF)*	PDFs (1)	PDF (18)	Y
*Proctolin*	Proctolin (1)	Proctolin (5)	N
*short neuropeptide Fs (sNPFs)*	sNPFs (4)^†^	sNPF-1 (11)	Y
		sNPF-2 (19)	Y
		sNPF-3 (6)	Y
		sNPF-4 (6)	Y
*Sulfakinin (SKs)*	SKs (3)^‡^	SK-0 (7-9)	Y/N
		SK-1 (9)	Y
*Drosophila tachykinin (Dtk)*	DTKs (6)	DTK-1 (10)	Y
		DTK-2 (9)	Y
		DTK-3 (9)	Y
		DTK-4 (10)	Y
		DTK-5 (15)	Y
		DTK-6 (9)	Y

*Ten (*D. mojavensis*), 11 (*D. ananassae*), 12 (*D. willistoni*), 14 (*D. erecta*), 17 (*D. grimshawi*), 13 (all other species). ^†^The processed forms sNPF-1^4-11 ^and sNPF-2^12-19 ^are typically sequence-identical. ^‡^Three SKs are predicted from the precursor. Only two have so far been biochemically demonstrated.

### Mass spectrometric characterization

In *Drosophila *larvae, the main neurohemal organs that store and release peptide hormones are the ring gland, and the thoracic and abdominal perisympathetic organs. The epitracheal cells (Inka cells) are endocrine glands along the trachea. These tissues represent a rich source of neuropeptides: their peptidome contains about half of all known *D. melanogaster *neuropeptides [35,36]. To biochemically assess whether the neuropeptides are expressed and processed as predicted, we directly profiled these neurohemal organs in *D. sechellia*, *D. pseudoobscura*, *D. mojavensis *and *D. virilis*. These species cover main phylogenetic lines within *Drosophila*. Obtained masses in the range of 850-2,500 Da were matched to the theoretical masses of predicted peptides (Table 2). This - and the observed tissue distribution - revealed that the peptidome of the investigated peptide release sites is identical in all species, at least in the mass range up to 2.5 kDa. In other words, all fruit flies appear to store the same set of (ortholog) peptides as *D. melanogaster *in the respective neurohemal release sites [35,36].

**Table 2:** Amino acid sequences and mono-isotopic masses of detected peptides

Peptide name	Species*	Gene^†^	Sequence	[M+H]^+^	Distribution^‡^
Adipokinetic hormones		CG1171			
AKH	1-5		pQLTFSPDWa	975.5	RG
AKH intermediate product	1-5		pQLTFSPDWGK-OH	1161.6	RG
CAPA peptides		CG15520			
CAPA-PVK-1	1-4		GANMGLYAFPRVa	1294.7	aDS
CAPA-PVK-1	5		GANMGLYTFPRVa	1324.7	aDS
CAPA-PVK-2	1-2		ASGLVAFPRVa	1015.6	aDS
CAPA-PVK-2	3		AGLVAFPRVa	928.6	aDS
CAPA-PVK-2	4		PGLVAFPRMa	986.6	aDS
CAPA-PVK-2	5		ASLVPFPRVa	984.6	aDS
CPPB	1-2		GDAELRKWAHLLALQQVLD	2176.2	RG, aDS
CPPB	3		SDAELRKFAHLLALQQVLD	2167.2	RG, aDS
CPPB	4		SESELRKWAHLLALQQALD	2208.2	RG, aDS
CPPB	5		SDSELRKWAHLLALQQALD	2194.2	RG, aDS
CAPA-PK	1-4		TGPSASSGLWFGPRLa	1531.8	aDS
CAPA-PK	5		TGPSASSGMWFGPRLa	1549.8	RG, aDS
CAPA-PK^2-15^	1-4		GPSASSGLWFGPRLa	1430.7	RG
CAPA-PK^2-15^	5		GPSASSGMWFGPRLa	1448.7	RG
Corazonin	1-5	CG3302	pQTFQYSRGWTNa	1369.6	RG
Corazonin^3-11^	1-5	CG3302	FQYSRGWTNa	1157.5	RG
Myosuppressin (MS)	1-5	CG6440	TDVDHVFLRFa	1247.6	RG
Eclosion-triggering hormones		CG18105			
ETH-1	1-2		DDSSPGFFLKITKNVPRLa	2033.1	PTC
ETH-1	3		DDSPGFFLKITKNVPRLa	1946.1	PTC
ETH-1	4-5		DESPGFFLKITKNVPRLa	1960.1	PTC
ETH-2	1-2		GENFAIKNLKTIPRIa	1713.0	PTC
ETH-2	3		SESFGMKNLKTIPRIa	1720.1	PTC
ETH-2	4		GEAFLMKNMKTIPRIa	1748.0	PTC
ETH-2	5		SEGFPMKNIKTIPRIa	1730.0	PTC
FMRFa-like peptides		CG2346			
FMRFa-2	1-5		DPKQDFMRFa	1182.6	tDS
FMRFa-2'	3		VPKQDFMRFa	1166.6	tDS
FMRFa-2"	5		APPSDFMRFa	1066.5	tDS
FMRFa-2"'	4		SPSDFMRFa	985.5	tDS
FMRFa-2""	3,5		APSDFMRFa	969.46	tDS
FMRFa-2""'	4-5		DPSQDFMRFa	1141.51	tDS
FMRFa-3	1-2		TPAEDFMRFa	1112.5	tDS
FMRFa-3'	3		TPSDFMRFa	999.5	tDS
FMRFa-4	1-2, 4-5		SDNFMRFa	915.4	tDS
FMRFa-4'	3		SDNFMRLa	881.4	tDS
FMRFa-5	1-5		SPKQDFMRFa	1154.6	tDS
FMRFa5 extended	1-2		SPHEELRSPKQDFMRFa	2003.0	tDS
FMRFa5 extended	3		SPQQELRSPKQDFMRFa	1993.0	tDS
FMRFa5 extended	4		NMNFHEELRSPKQDFMRFa	2325.1	tDS
FMRFa5 extended	5		NLNFHEELRSPKQDFMRFa	2307.1	tDS
FMRFa-6	1-5		PDNFMRFa	925.4	tDS
FMRFa-7	1-2		SAPQDFVRSa	1005.5	tDS
FMRFa-7'	3		SAPPEFERYa	1094.5	tDS
FMRFa-7"	4		AAPSDFERFa	1038.5	tDS
FMRFa-7"'	5		SAPTEFERNa	1049.5	tDS
FMRFa-8	1-2		MDSNFIRFa	1028.5	tDS
FMRFa-8'	3-5		MDSNFMRFa	1046.5	tDS
HUGIN-pyrokinin					
HUG-PK	1-5	CG6371	SVPFKPRLa	942.6	RG
IPNa	1-2	CG3441	NVGTLARDFQLPIPNa	1653.9	VG
IPNa	3		NVGTLARDFQLPMPNa	1671.9	VG
IPNa	4-5		NVGTLARDFQLPNa	1443.8	VG
leucokinin	1-5	CG13480	NSVVLGKKQRFHSWGa	1743.0	VG
sNPF		CG13968			
sNPF-1^4-11^	1-5		SPSLRLRFa	974.6	RG
sNPF-2^12-19^	1-3, 5		SPSLRLRFa	974.6	RG
sNPF-2^12-19^	4		SPSMRLRFa	992.6	RG
sNPF-1	1-2		AQRSPSLRLRFa	1329.8	RG

*Drosophila species: 1, *melanogaster*; 2, *sechellia*; 3, *pseudoobscura*; 4, *mojavensis*; 5, *virilis*. Data for *D. melanogaster *are from [35,36]. ^†^BDGP gene annotation for *D. melanogaster*. ^‡^aDS, abdominal dorsal sheath; PTC, peritracheal cells; RG, ring gland; tDS, thoracic dorsal sheath; VG, ventral ganglion.

#### Direct mass spectrometric profiling of the ring gland

**Figure 2:**
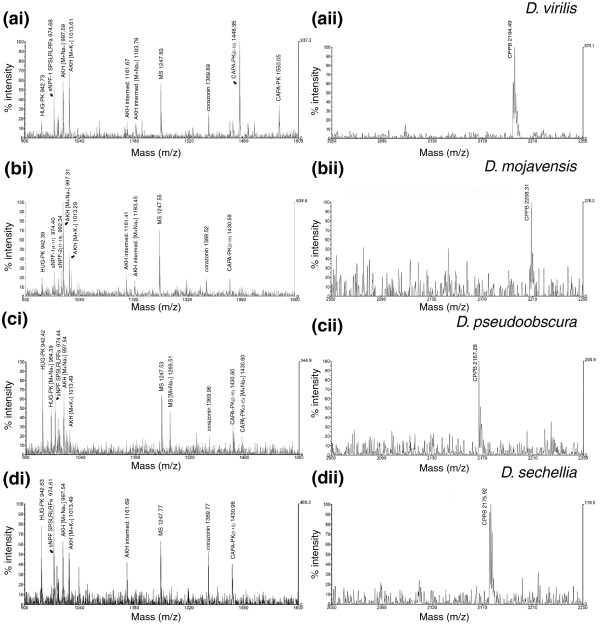
Direct peptide profiling of the ring gland of different *Drosophila *species. **(ai-di) **Mass range 900-1,600 Da. The protonated mass of AKH is not visible, but the Na^+ ^and K^+ ^adducts are prominent. **(aii-dii) **Mass range 2,050-2,250 Da. Only one mass peak corresponding to CPPB is visible.

The ring gland contained the adipokinetic hormone (AKH; pQLTFSPDWa), the AKH processing intermediate pQLTFSPDWGK, myosuppressin (MS), corazonin, corazonin^3-11^, the pyrokinins CAPA-PK^2-15 ^and hugin (HUG)-PK, and the CAPA precursor peptide B (CPPB) (Figure 2 and Additional data file 3). As in *D. melanogaster*, the mass peak at 974.6 Da indicates the presence of SPSLRLRFa in *D. sechellia*, *D. pseudoobscura*, and *D. virilis*. The origin of SPSLRLRFa in these species is ambiguous, since it could represent short neuropeptide F (sNPF)-1^4-11 ^or its sequence-identical paralog sNPF-2^12-19^. The finding of mass peaks at 974.6 and 992.6 in ring gland profiles of *D. mojavensis *- corresponding to sNPF^4-11 ^and the aberrant *D. mojavensis *sNPF-2^12-19 ^SPSMRLRFa - indicates that, in fact, both sNPF-1^4-11^ and sNPF-2^12-19 ^occur in the ring gland of *Drosophila *species. In *D. sechellia*, a mass peak corresponding to the full sNPF-1 was found in one preparation.

#### Direct mass spectrometric profiling of neurohemal release sites in the ventral ganglion

**Figure 3:**
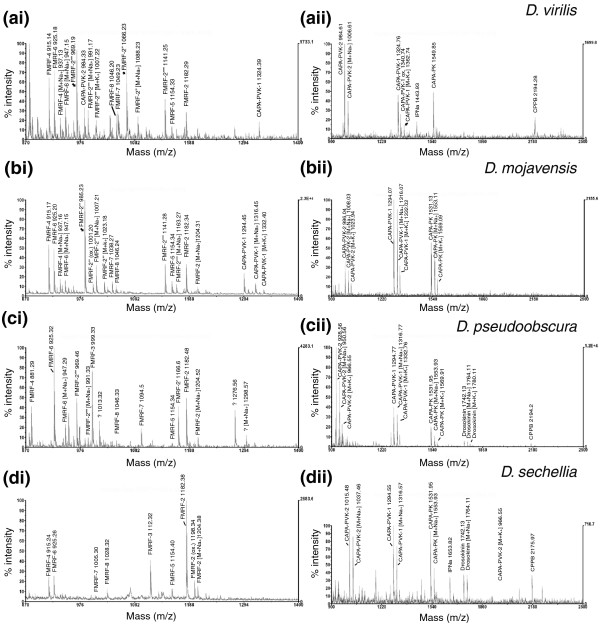
Direct peptide profiling of the dorsal neural sheath of different *Drosophila *species. **(ai-di) **Thoracic portion containing FMRFa-like peptides. Note that peak intensity corresponds with isocopy number in the FMRFa prepropeptide. Small peaks corresponding to CAPA peptides from overlapping Va neurites are visible in *D. virilis *and *D. mojavensis*. **(aii-dii) **Abdominal portion containing CAPA peptides. Peaks corresponding to drosokinin and IPNa in *D. pseudoobscura *and *D. sechellia *represent contaminations with ganglionic neurites or the segmental nerve.

The neurohemal organs of the ventral ganglion are the thoracic and abdominal perisympathetic organs. In *Drosophila *and other flies, these organs persist during the larval stages but are subsequently reduced during pupal metamorphosis. In the adult fly, the innervating peptidergic neurites supply a neurohemal zone directly below the dorsal neural sheath [38,39]. Since we did not succeed to specifically dissect the tiny larval perisympathetic organs, we directly profiled adult dorsal neural sheath preparations that were carefully cleaned of attached nervous tissue (n = 5-9 for each species). As in *D. melanogaster *[35], preparations from thoracic portions of the dorsal neural sheath contained the FMRFa-like peptides of the FMRF-prepropeptide (Figure 3ai-di). Preparations from abdominal portions contained the CAPA peptides CAPA-PVK-1 and -2, CAPA-PK and CPPB (Figure 3aii-dii). Occasionally, mass peaks corresponding to CAPA peptides were found in thoracic preparations, and FMRFa-like peptides in abdominal preparations. This corresponds to the variable extent of overlap of the more posterior CAPA neuron projections with more anterior FMRFa-like peptide neuron projections. Concomitantly, mass spectra from intermediate portions of the dorsal neural sheath consistently showed both CAPA and FMRFa-like peptide peaks.

In each species, the masses of all predicted FMRFa-like peptides of the FMRF-prepropeptide could be detected (Table 2) with the exception of FMRFa-1. This peptide invariantly has the carboxy-terminal sequence FMHFa in the investigated species, and thereby lacks the easily protonated Arg that makes FMRFa-1 difficult to detect in peptide mixtures by the MALDI process [35,40]. In many FMRFa-like peptide-containing spectra, a mass peak around 2 kDa was prominent. In each species, this mass peak matched the theoretical mass of the respective extended form of FMRFa-5 (FMRFa-5^ext^), which would result from prohormone cleavage of FMRF-4 and FMRF-6 without internal cleavage of the single Arg cleavage site of FMRF-5 (Additional data file 4). An extended form of FMRFa-5 had not been described from *D. melanogaster*. We therefore reviewed our old data from *D. melanogaster *larvae [36]. In many spectra, we found a distinct mass peak at 2,003.0 Da, which matches the theoretical mass of FMRF-5^ext ^of *D. melanogaster *but was previously overlooked. The consistent occurrence of prominent mass peaks corresponding to the theoretical mass of FMRF-5^ext ^in the different *Drosophila *species is unlikely to have occurred by chance, and therefore indicates a new processing product of the *Drosophila *FMRFa precursor. It is unclear whether FMRF-5^ext ^is released as a peptide hormone, or only represents a processing intermediate.

Besides CAPA- and FMRFa-like peptides, mass peaks corresponding to leucokinin and IPNa were occasionally detected in dorsal neural sheath preparations (Figure 3). Leucokinin and IPNa are dominant peptides in ventral ganglion preparations [35] and likely represent a contamination of the dorsal neural sheath by adhering peptidergic neurites.

#### Direct mass spectrometric profiling of the peritracheal cells

**Figure 4:**
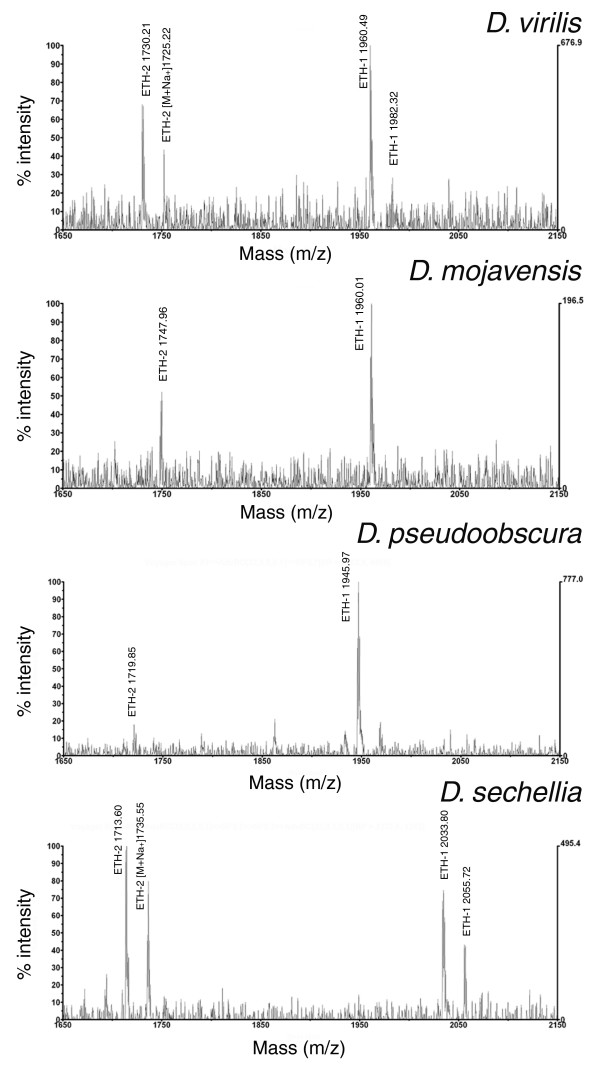
Direct peptide profiling of tracheal preparations containing the peritracheal cells of different *Drosophila *species. Peaks corresponding to the [M+H]^+ ^or [M+Na]^+ ^adducts of the two ETHs are visible besides the typical and possibly non-peptidergic tracheal peaks [22].

The larval peritracheal cells are located at stereotypic locations near the primary branchings of trachea from the main trunk [41]. As in *D. melanogaster*, spectra obtained with the laser beam directed at these branching sites consistently showed mass peaks corresponding to ecdysis-triggering hormone (ETH)-1 and -2 in all species (Figure 4). The mass of ETH-1 was detected in 8 out of 15 preparations in *D. virilis*, in 9 out of 11 preparations in *D. mojavensis*, in 12 out of 13 preparations in *D. pseudoobscura*, and 4 out of 6 preparations in *D. sechellia*. Equivalent numbers for ETH-2 were 11/15, 6/11, 7/13 and 6/6.

### Peptide copy numbers

Alignment of the prepropeptide sequences showed that the peptide families of each *Drosophila *species consist of an identical set and number of ortholog neuropeptide copies, with the exception of FMRFa-like peptides (Table 1; Additional data files 1 and 2). For example, in all species the crustacean cardioactive peptide (CCAP) precursor contains one CCAP, and the allatostatin A (ASTa) precursor contains 4 ASTa peptides. The FMRFa precursor, however, encodes 10 FMRFa-like peptides in *D. mojavensis *and *D. virilis*, 11 FMRFa-like peptides in *D. ananassae*, 12 FMRFa-like peptides in *D. willistoni*, 14 FMRFa-like peptides in *D. erecta*, 17 FMRFa-like peptides in *D. grimshawi*, and 13 FMRFa-like peptides in all other species. The *fmrf *gene contains 2 exons, of which exon II codes for the whole FMRFa prepropeptide. The differences in peptide-coding sequences can thus not be explained by exon duplication. Higher numbers of tandem repeats exist for FMRFa-2 (DPKQDMRFa; for example, 5 copies in *D. melanogaster*, 7 copies in *D. grimshawi*) in all species but *D. mojavensis *and *D. virilis*. This may suggest that mispairing of template versus replicating nucleotide sequences coding for this peptide has resulted in insertions/deletions during *Drosophila *evolution and has caused the high number of FMRFa-like peptide copies.

Two prepropeptides contain neuropeptides that are usually not grouped into the same peptide family: the CAPA prepropeptide contains two periviscerokinins and one pyrokinin, and the neuropeptide-like precursor (NPLP)1 prepropeptide contains one MTYamide, one IPNamide and one non-amidated peptide. The CAPA pyrokinin and the NPLP1 peptides have therefore been treated as single copy peptides (but see Discussion).

### Peptide-coding sequences are more conserved than spacer sequences

**Figure 5:**
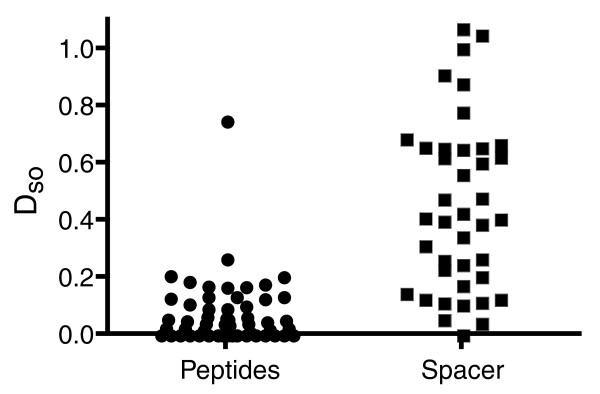
Plot of the average distance between orthocopies and ortholog spacers. Each data point represents the average amino acid distance D_so _between orthocopies or ortholog spacer regions. With the exception of FMRFa-7, the peptide orthocopy distances have values below 0.3 and do not follow a Poisson distribution as is seen for the spacers.

If the neuropeptide sequences are subjected to stabilizing selection due to their signaling function, it is reasonable to assume that the peptide-coding parts of the prepropeptides are more conserved than the spacers (the parts separating the bioactive peptides), which by existing evidence do not act as signaling molecules in insects. In other words, the sequence similarity between ortholog neuropeptide parts of the prepropeptides is likely to be higher than the sequence similarity of ortholog spacer parts. To test this hypothesis, it is not sufficient to simply calculate amino acid identities, since substitutions of amino acids do not occur randomly but are correlated with their physico-chemical characteristics [42]. We thus calculated the overall average amino acid distance D_so _for each set of orthologs (Figure 5) based on the Jones-Thornton-Taylor (JTT) matrix [43] as a more appropriate measure of sequence variation (see Material and methods). The raw values are listed in Additional data file 5. The median D_so _between peptide orthologs was 0.041, and thus significantly lower than the calculated 0.408 for the spacers (Figure 5; Mann-Whitney, two-tailed *p *< 0.0001, U = 211.5), although the sequence of several spacers was quite conserved (for example, in the CCAP or CAPA prepropeptides). In contrast to the peptides (*p *< 0.01), the spacer distances followed a Poisson distribution.

A closer look at the data (Additional data file 5) shows that high D_so _values only occur in multiple copy peptide families. For example, neuropeptide F (NPF) and MTYa show the highest D_so _for single copy families (0.134 and 0.093, respectively). The respective maximum values for multiple copy families are 0.748 for FMRFa-7, 0.601 for sulfakinin (SK)-0, 0.267 for ETH-2, 0.208 for FMRFa-7, and 0.205 for CAPA-PVK-2. Yet, orthocopy sets without sequence variation or with low D_so _values occur not only in single copy, but also in each multiple copy peptide family: CAPA-PVK-1 (0.042), ETH-1 (0.025), SK1- (0), ASTa-3 and -4 (0), sNPF-1 (0), myoinhibiting peptide (MIP)-3 and -4 (0), *Drosophila *tachykinin (DTK)-3 (0.02) and FMRFa-2 and -6 (0).

### The average distance between all peptides in a family is higher for families with multiple paracopies

**Figure 6:**
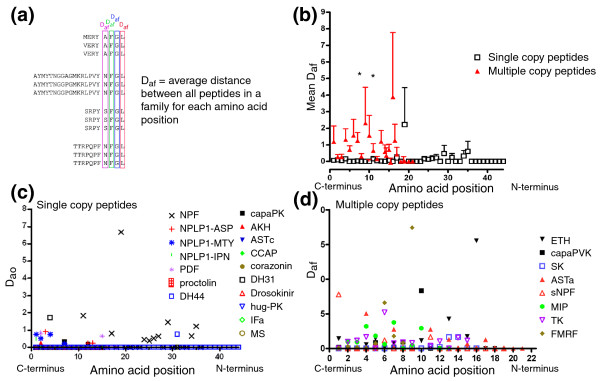
Plot of the average distance between all paracopies in a family. Each data point represents the average amino acid distance D_af _between all paracopies of a peptide family for each amino acid position throughout the species as outlined in **(a)**. **(b-d) **The mean ± standard deviation of the data (b) for single copy peptides (c) and multiple copy peptide families (d). Paracopy number is color-coded in (d): black, 2; blue, 3; red, 4; green, 5; purple, 6; and brown, 10-17. The asterisks indicate a significant difference between multiple and single copy peptides.

To test for differences in the sequence variability between single and multiple copy peptide families, we computed the average amino acid distance D_af _for each amino acid position between all paracopies within a peptide family (Figure 6a, d) and then calculated the mean (Figure 6b). For single copy peptides, we calculated the corresponding average amino acid distance D_ao _for the respective orthologs (Figure 6c). The results in Figure 6 show that the mean D_af _between paracopies of multiple copy peptide families is typically higher than the D_ao _observed between the single copy peptides. Due to a large standard variation, these differences are only significant for amino acid positions 5 and 7 from the carboxyl terminus (paired *t*-test, *p *< 0.05). This reflects the spread of sequence variation in multiple copy peptide families. For most amino acid positions there are families that show no variation, and, at the same time, families with considerable sequence variation. The high mean D_ap _at position 1 from the carboxyl terminus mostly originates from the sNPFs, which end either RFa (sNPF-1 and -2) or RWa (sNPF-3 and -4). There is no clear tendency that the sequence variation increases from the carboxyl to the amino terminus; a correlation between D_af _and copy number is not discernible (Figure 6d).

### Orthologs of single and multiple copy peptide families are equally sequence-conserved

**Figure 7:**
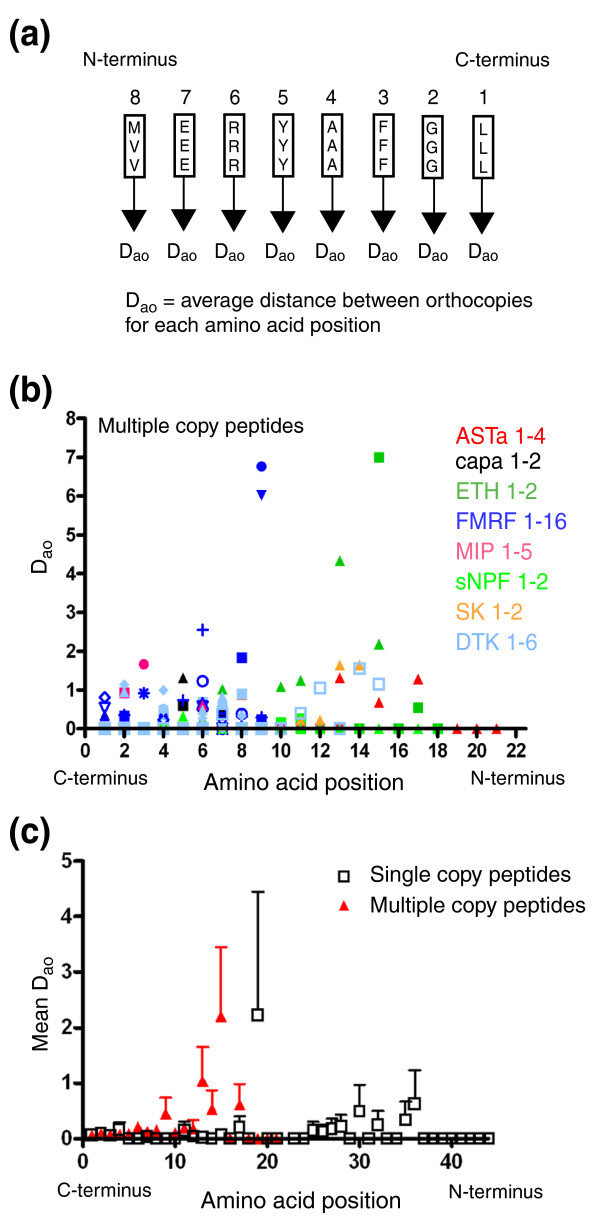
Plot of the average distance between orthocopies for each amino acid position. Each data point represents the average amino acid distance D_ao _between the orthocopies for each amino acid position throughout the species as outlined in **(a)**. **(b) **The D_ao _for multiple copy peptide families. **(c) **The mean D_ao _± standard deviation for single (black) and multiple (red) copy peptide families (see Figure 6c). The different shapes code for paracopy numbers: filled square, 1; filled triangle, 2; inverted filled triangle, 3; filled diamond, 4; filled circle, 5; open square, 6; open triangle, 7; open triangle, 8; open diamond, 9; open circle, 10; cross, 11; plus sign, 12; asterisk, 13.

The distance D_af _contains both the sequence variation between individual orthologs (inter-ortholog variation) as well as between individual paracopies (inter-paracopy variation; Figure 1). To test the contribution of the inter-ortholog variation to D_af_, we calculated the average amino acid distance D_ao _for each amino acid position for each set of orthocopies individually (Figure 7). A comparison of Figures 7c and 6b shows that the mean D_ao _for the ten carboxy-terminal amino acids is considerably smaller than the mean D_af _and not significantly different between single and multiple copy peptides. This region likely contains the active core of the peptides, which typically consists of the last five to seven carboxy-terminal amino acids and the amidation signal (for example, [44-47]). Somewhat higher D_ao _values occurred for more amino-terminal amino acids. This shows that the orthologs are strongly sequence-conserved throughout *Drosophila*, irrespective of whether they belong to a single or multiple copy peptide family - with the exception of FMRFa-7 and SK-0 (see below).

### Sequence variation mostly originates from sequence variation between paracopies

**Figure 8:**
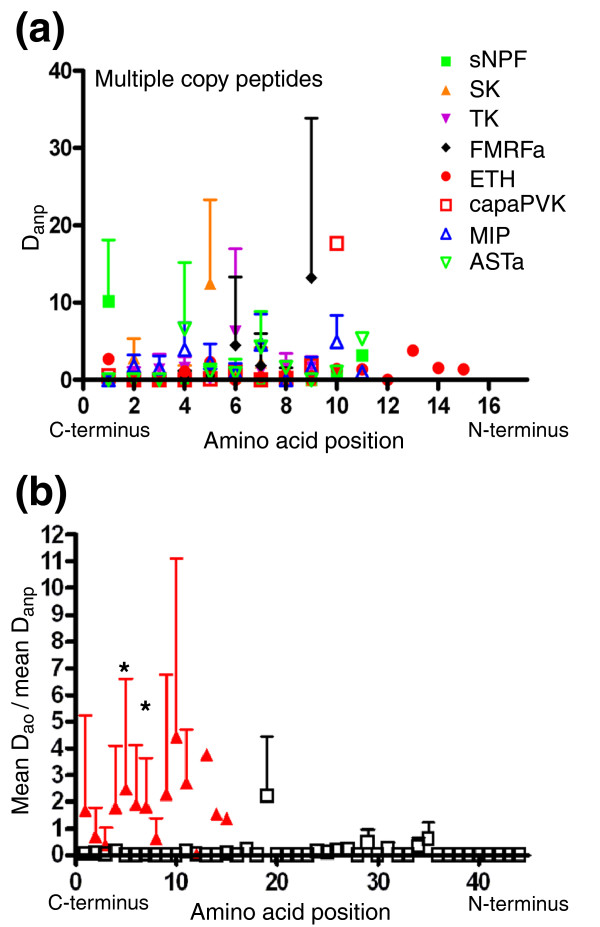
Plot of the net distance between paracopies for each amino acid position. **(a) **Each data point represents the average net amino acid distance D_anp _± standard deviation between the paracopies for each amino acid position throughout the species. **(b) **The mean ± standard deviation of the data for multiple copy peptides compared to the mean D_ao _± standard deviation of single copy peptides (see Figure 6c). The asterisks indicate a significant difference between multiple and single copy peptides.

We next calculated the average net amino acid distances (Figure 1) between paracopies D_anp _for multiple copy peptide families; results are shown in Figure 8. The mean D_anp _was higher than the mean D_ao _of single (Figure 8b) or multiple copy (compare Figure 7c) peptides throughout amino acid position 1-11. This was again only significant for positions 5 and 7 due to the high standard variations (paired *t*-test, *p *< 0.05). As for D_af_, the high variation of D_anp _reflects the spread of the degree in sequence variation between multiple copy peptide families: given positions were variable in some peptide families, but imvariantly occupied by the same amino acid in others. The high D_anp _of 1.69 at position 1 from the carboxyl terminus is again caused by the carboxy-terminal difference RFa and RWa between the sNPFs. When omitting the RWamides sNPF-3 and -4 - which could not be biochemically detected yet - this value drops to 0.42. With this value, it seems that the amino acids at positions 1-3 and 8 from the carboxyl terminus are the most conserved amino acids between the paracopies of each multiple copy peptide family.

### Sequence variation is not related to receptor number

**Figure 9:**
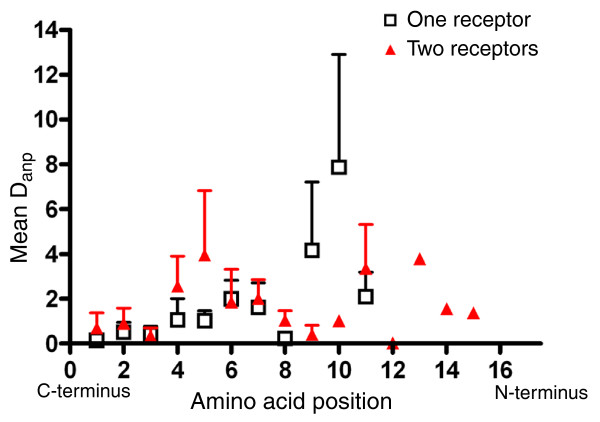
Plot of the net distance between paracopies for each amino acid position. Each data point represents the average net amino acid distance D_anp _± standard deviation between the paracopies for each amino acid position throughout the species for peptide families with one (open black squares) or two (closed red triangles) known receptors.

The majority of G protein-coupled peptide receptors of *D. melanogaster *have been deorphanized, with some still uncharacterized to date [48]. From the obtainable literature [48-51], we compiled the number of characterized *D. melanogaster *G protein-coupled receptors per peptide family. Although these numbers may be subjected to future changes, to date there are either only one or two receptors known for the paracopies of each peptide family. The occurrence of two receptors for some peptide family opens the possibility for receptor-ligand coevolution and subsequent sub- and neofunctionalization of paracopies. To test for this, we plotted the D_anp _between multiple copy peptide families with one known receptor against those with two known receptors. Although there are differences (Figure 9), they are neither statistically significant nor do they follow an obvious pattern. This result speaks against a sub- or neofunctionalization of paracopies during the evolution of *Drosophila*.

## Discussion

We datamined the 11 new *Drosophila *genomes for homologs of the 22 described prepropeptide genes of *D. melanogaster *encoding neuropeptides up to a length of 50 amino acids [52,53]. From these data, we were able to predict 53-60 neuropeptides for each species. These peptides are known or are likely to signal via G protein-coupled receptors [48]. Larger protein hormones (>50 amino acids) have not been included, because they are expected to have a smaller proportion of residues that are important for their pharmacological efficacy (see, for example, [54]), which makes it difficult to directly compare their sequence variability to that of the smaller neuropeptides.

### Accuracy of peptide predictions

The obtained mass fingerprints of the neurohemal organs and endocrine cells were identical: in each species, the obtained masses corresponded to the respective orthologs in *D. melanogaster *neurohemal organs or peritracheal cells [35,36]. Vice versa, for each peptide characterized in the neurohemal organs or peritracheal cells of *D. melanogaster*, there was a mass present that corresponded to the respective ortholog in the other species. Similar to the use of peptide fingerprints in proteomics, the exactly matching tissue fingerprints chemically identify the underlying peptides and precursor products with high probability. All fingerprint masses matched the respective theoretical masses calculated for the *in silico *predicted peptides. In conclusion, the mass spectrometric profiling supports our *in silico *prediction of the neuropeptidome.

### The peptidome is evolutionarily conserved throughout the genus *Drosophila*

The finding of identical peptide hormone complements in the mass range of 800-2,500 Da in main *Drosophila *phylogenetic lineages suggests that the peptidome of the major neurohemal organs and the peritracheal cells has been evolutionary stable for at least 40-60 million years since the divergence of the *Drosophila *species from their last common ancestor. Obviously, all *Drosophila *species share the same peptidergic hormonal communication possibilities. Even more, our genomic comparisons suggest that the whole peptidome is highly conserved throughout the genus *Drosophila*. We observed no loss of peptide precursors, or individual peptides as suggested to have occurred, for example, between flies and mosquitoes [55]. Thus, the number of peptide copies in each precursor was identical throughout the species with exception for the FMRFamides, which most likely duplicated by unequal recombination. These recombination events must have occurred independently from each other, since multiple repeats of FMRFa-2 coding sequences are present both in the *Sophophora *subgroup and the Hawaiian species of the *Drosophila *subgroup (*D. grimshawi*), but are lacking in the other *Drosophila *subgroup species *D. virilis *and *D. mojavensis*. Unequal recombination is also the likely mechanism behind the duplication of most other multiple copy peptides, but for them recombination must have occurred prior to *Drosophila *speciation.

The high conservancy of the peptidome is remarkable and unexpected, since drosophilid flies have undergone several radiations and have adapted to a variety of environments with, for example, a very different supply of water, such as sea shores, forests and deserts [34,56]. In contrast, the genome of the tenebrionid beetle *Tribolium castaneum *shows a gene expansion for putative diuretic peptides [57]. This has been interpreted as an adaptation to dry conditions in tenebrionids, a beetle family that thrives in deserts and other very dry places. It is, however, unclear whether this is a special tenebrionid or a common beetle feature. At least for fruit flies, our data show that the adaptation to different environments is not paralleled by changes in the number or increased sequence variability of diuretic hormones or other neuropeptides.

### Neuropeptide sequences are subjected to stabilizing selection

In our analysis, spacer sequences showed significantly higher amino acid distances than peptide sequences. This suggests that *Drosophila *neuropeptides are subjected to stabilizing selection or evolutionary constraint to a much larger extent than spacer sequences. This is further supported by the non-random distribution of peptide distances not observed for spacers. This finding may not be unexpected, but is shown here for the first time on a neuropeptidome level.

In *Drosophila*, there is a higher proportion of highly constrained codons in essential genes than in any other dispensability class [58]. As hypothesized at the outset, stabilizing selection and the resulting sequence conservation may thus indicate functional importance of neuropeptides, signaling molecules for which single amino acid exchanges can result in drastically altered receptor efficacy, binding or effect (for example, [28,59]). If this hypothesis is correct, then the observed low inter-orthocopy distances (D_ao_, D_so_) indicate that the multiple peptide copies are functionally important and not individually dispensable.

The observed higher amino acid distances that reflect a considerable sequence variation for the spacers do not allow us to conclude that structural features of the spacers are unimportant for proper peptide processing and packaging into secretory vesicles. They speak, however, against a general signaling function of the spacer regions ('associated peptides') at the receptor binding site, where single amino acid changes can already result in altered efficacy, effect or specificity (for example, [28,59]). Nevertheless, this conclusion needs proper physiological testing. Several spacer regions are quite conserved throughout the *Drosophila *species (for example, in the CAPA and CCAP precursor), and a FMRFa-spacer-derived peptide has been shown to modulate the activity of FMRFa at the receptor in *Lymnea *[60].

### Peptide copies are unlikely to have undergone a phase of neutral mutation

The comparably high D_anp _distances show that there is sequence-variation between paracopies (inter-paracopy variation). Assuming that paracopies at some point have arisen from a common ancestor, we have hypothesized at the outset that newly duplicated paracopies can escape selection pressure and may be allowed to drift neutrally. However, the small D_ao _distances between orthocopies (inter-orthocopy variation) do not support this scenario. A significant difference in sequence variation between the individual sets of orthocopies was not observed between single and multiple copy peptide families, and inter-orthocopy distances were small compared to the distances found between spacer regions. This suggests that: the inter-paracopy variation originates from a time before divergence of the *Drosophila *taxa; and paracopies have never fully escaped selection pressure and have never experienced a phase of neutral mutation. Hence, the classic theory for duplicated genes may only apply in a limited sense for paracopies.

At the outset, we reasoned that peptide copies following the 'more-of-the-same' concept will show low sequence variation between paracopies. For paracopies with differential activities, we expected at the same time an increased sequence variation between paracopies. Since the inter-paracopy distances D_anp _were similar for all multiple copy peptide families and did not correlate with receptor number, it is not possible to draw conclusions from our data in this respect. For *Drosophila*, there are also no data regarding different half-lives during peptide degradation, or induction of ligand-selective receptor conformation and activity [61] as has been demonstrated for locust and cockroach AKHs [27,28].

The CAPA and NPLP1 prepropeptides contain neuropeptides that are usually not grouped into the same peptide family. We have therefore treated the CAPA pyrokinin and the NPLP1 peptides as single copy peptides. However, some sequence similarities can be found between CAPA pyrokinins and periviscerokinins, and between the amino-terminal stretches of the NPLP1 peptides [62]. It has also been shown that the CAPA pyrokinin specifically activates a G protein-coupled receptor (CG9918) that is evolutionarily related to the other *Drosophila *pyrokinin receptors, but is not activated by CAPA periviscerokinins and the HUG-pyrokinin at physiological concentrations [63]; data on NPLP1 peptide receptors are not yet available. Thus, it is possible that at least the CAPA peptides are, in fact, an example of multiple copies that have sub- or neofunctionalized by acquiring sequence variation: the CAPA prepropeptide appears to date back at least to the origin of insects, since it contains a few periviscerokinins plus one highly sequence-conserved pyrokinin in all insect taxa investigated so far [64]. If this is the case, this sub- or neofunctionalization must have occurred a long time before the radiation of *Drosophila*. While this justifies the classification of at least the CAPA pyrokinin as a single copy peptide in this study, it emphasizes the need for further comparisons similar to that reported here for *Drosophila *on larger phylogenetic units spanning longer evolutionary time frames. Such studies will soon become possible with the increasing number of fully sequenced insect genomes.

### The calculated distances correlate with pharmacological efficacy

Although the inter-orthocopy distance D_so _was, in general, very low throughout the peptides, we observed differences in D_so _within the CAPA-PVK, ETH, FMRFamide and tachykinin peptide families. Can these differences be correlated to differential activities? A comparison of the calculated D_so _values with the available pharmacological data shows that there is at least a correlation to the reported efficacies: the paracopies with lower amino acid distance typically are the ones with higher receptor or pharmacological activity.

For the ETHs, the EC_50 _of the paracopy with the lowest sequence variation (ETH-1) is nine times more potent in heterologous receptor assays than the more sequence-variable ETH-2 [65,66]. Two groups have characterized the FMRFa receptor of *D. melanogaster *in heterologous expression systems. Both found that FMRFa-6 (PDNFMRFa) has the highest potency to activate the FMRFa receptor, whereas FMRFa-7 has no activity at all [67,68]. Meeusen and colleagues [68] report similar EC_50 _values for FMRFa-2 to -5. All were only slightly less potent than FMRFa-6. Cazzamali and Grimmelikhuijzen [67] found the following order: FMRFa-6 > FMRFa-2 > FMRFa-3,-5,-8 > FMRFa-1 > FMRFa-4. This pharmacological ranking correlates well with the calculated D_so _distances (in brackets): FMRFa-6 = FMRFa-2 (0) < FMRFa-5 (0.04) < FMRFa-8 (0.064) < FMRFa-4 (0.11) < FMRFa-1 (0.127) < FMRFa-3 (0.172) << FMRFa-7 (0.748).

For the DTKs, our data predict the following ranking of pharmacological activity: DTK-3 > DTK-1 > DTK-6 > DTK4 = DTK-5 > DTK-2. This again corresponds quite well with the efficacy of DTKs on DTKR - one of the two DTK receptors known - in HEK-293 cells [69]: DTK-1 > DTK-3 = DTK-6 > DTK-4 > DTK-5 > DTK-2. For CAPA-PVKs, the copy with the lower sequence variation (CAPA-PVK-1) is more effective in inducing calcium responses and fluid transport in the Malpighian tubules [70] and about 1.5-times more potent in receptor assays than the more sequence variable CAPA-PVK-2 [65,71]. In other words, the more potent peptides were typically the more sequence-conserved. This might extend well beyond *Drosophila*. For example, CAPA-PVK-2 orthologs are much more sequence-variable in their carboxyl terminus than CAPA-PVK-1 orthologs not only in *Drosophila*, but also in other flies [72,73]. At the same time, the housefly *Musca domestica *CAPA-PVK-2 shows a ten-times diminished efficacy in fluid secretion assays on housefly Malpighian tubules compared to *M. domestica *CAPA-PVK-1 [45]. The degree of sequence variation appears not to be linked with peptide position along the precursor.

In contrast to the CAPA-PVKs, ETHs, FMRFamides and DTKs, the SK-1 and -2, MIP and ASTa copies all showed a consistently low inter-orthocopy distance. The pharmacological profiles of SK and MIP copies on their respective receptors have not been characterized so far, but such data exist for the two *Drosophila *ASTa receptors (DARs) expressed in Chinese hamster ovary (CHO) cells [74,75]. The data suggest that DAR-1 has a lower sensitivity for ASTa-4 than for ASTa-1 to -3, whereas DAR-2 is more sensitive to ASTa-3 to -4. It has, however, to be kept in mind that not all peptide receptors have been deorphanized to date, and that signaling properties and specificities of receptors may be changed by modifying proteins such as RAMP or RGS in native cells. It is also possible that ligand-selective receptor conformations may exist [61], and the ligand properties and activated intracellular pathways *in vivo *may be different to those in heterologous expression systems. This may explain why - in contrast to the data from heterologous expression systems - all FMRFamides had a similar dose-response effect at the neuromuscular junction [22]. Only FMRFa-7 (SAPQDFVRSa) was inactive in all systems. Clearly, further data, especially from bioassays, will be needed to confirm the observed correlations between sequence variation and efficacy.

The evolution of neuropeptides and their receptors is linked, and neuropeptide receptors are under evolutionary pressure to maintain a high affinity to the authentic ligands [9,59]. The finding that the more sequence-variable neuropeptides typically had a lower pharmacological efficacy does not speak for the occurrence of fast adaptive structural changes of G protein-coupled receptors to maintain a high ligand affinity to sequence-variable peptides during the evolution of *Drosophila*. Does that mean that peptide copies with high amino acid distances are functionally unimportant? The by far highest distances were found for FMRFa-7 (0.748) and SK-0 (0.601). The carboxyl terminus of FMRFa-7 (FVRSa) is highly deviated and is the likely cause for its lack of receptor activation and bioactivity [22,67] (and see above). The high D_so _value of FMRFa-7 is around the mean value found for spacer regions, which suggests that FMRFa-7 has escaped selection pressure to a considerable amount. The high D_so _value for SK-0 correlates with its inactivity at the DSK-R1 receptor [76] and its lack of bioactivity at physiological concentrations below 1 μM [77]. Unlike SK-1 and -2, SK-0 has, furthermore, not been found biochemically so far [35,37]. Hugin-gamma, a predicted but obviously not processed peptide [78] that seems to be missing from the genome of *D. persimilis *[55], shows a D_so _of 0.259. Nevertheless, the synthetic *D. melanogaster *HUG-gamma is still able to activate the receptor [65,79]. We propose from this as a rough estimate that the peptides with an amino acid distance above 0.6 are nonfunctionalized. Distances below 0.3 and the non-Gaussian distribution of all other peptide copies suggest that they are under stabilizing selection that prevents nonfunctionalization by random or deleterious mutations.

## Conclusion

Taken together, our data provide evidence that the peptidome and the neuropeptide hormone complement has been conserved during the evolution of *Drosophila*, and shows that multiple peptide copies with biological activity are under stabilizing selection. Sequence conservation largely correlates with pharmacological activity. While all this suggest that multiple peptide copies are functionally important, it remains unclear why paracopies are under stabilizing selection.

It has to be stressed that our data are based on only a relatively small number of data points. This was unavoidable by the simple fact that further multiple copy neuropeptide families in *Drosophila *have not been identified. Consequently, our conclusions will need further validation and we hope that our work will provoke further studies on new data from the rapidly increasing number of genome projects. Our study emphasizes the value of these genome projects, and stresses the need for more comprehensive structure-activity studies and pharmacological characterization of peptides both in receptor and bioassays.

## Materials and methods

### Flies

*D. virilis *was obtained from a colony in Ulm. Genome library strains of *D. mojavensis*,*D. pseudoobscura*, and *D. sechellia *were obtained from the Tucson *Drosophila *Stock Center. Flies were kept at standard conditions - a light:day cycle of 12 h:12 h and either 18°C or 25°C. *D. virilis *and *D. sechellia *were raised on standard cornmeal medium, *D. mojavensis *and *D. pseudoobscura *on standard banana-*Opuntia *medium.

### Database searches

Peptide precursor genes were identified by tblastn homology searches against the respective *D. melanogaster *coding sequences using the PAM30 matrix of the *Drosophila *species BLAST site [80]. The coding sequences were identified and translated with GENSCAN [81] and compared with the GLEAN-predicted sequences in the databank. Amino acid sequences were aligned with the ClustalW algorithm implemented in MEGA3.1 [82] and plotted using GeneDoc [83]. Signal peptides were predicted by SignalP 3.0 [84]. Mono-isotopic masses of the predicted bioactive neuropeptides were calculated with Data Explorer 4.0 software (Applied Biosystems, Darmstadt, Germany).

### Peptide prediction

We predicted the processed bioactive peptides based on cleavage site consensus sequences [85] and comparison with the chemically characterized processing products from *D. melanogaster *[35-37]. Mono-isotopic masses were calculated for all peptides and listed for each species. Peptide designations were inferred from prepropeptide alignment with the ortholog *D. melanogaster *sequence, for example, the myoinhibiting peptide encoded on the *Mip *orthologs that aligned with *D. melanogaster *MIP-3 was also named MIP-3. This allows easy identification and reference to ortholog peptides. In the *fmrf*-precursor of *D. melanogaster*, several paracopies are sequence-identical and named either FMRFa-2 or -3. These paracopies and their orthologs were designated according to their position on the gene as FMRFa-2', FMRFa-2", and so on.

### Calculations of sequence variation/amino acid distances

The parts of the prepropeptides between the signal peptide and the bioactive peptide sequences flanked by the mono- or dibasic cleavage sites were assigned as spacers. For distance calculations of peptides, sequences were aligned from their carboxyl terminus. Gaps that occurred due to variable peptide copy length were deleted pairwise. Spacers were aligned by the ClustalW algorithm prior to distance calculation. Average distances were calculated as absolute values in MEGA3.1 for pairwise comparisons as outlined in Figure 1 by iterative procedures under a maximum likelihood formulation using the JTT matrix [43,82]. The JTT matrix was calculated from data of the Swiss-Prot protein sequence database and gives a measure of the probability that a given amino acid *i *is being replaced by residue *j *per occurrence of *j *[43]. Variable mutation rates among sites were assumed. Since the peptide sequences are too short to reliably estimate gamma parameters, we adopted a gamma distance with α = 2.4, which is very close to the true distance for sequence divergence under the JTT model [86]. Data were processed and plotted using Microsoft Excel and GraphPad Prism 4.0 (GraphPad Software, San Diego, CA, USA).

### Sample preparation

The central nervous system was dissected free from all surrounding tissue in standard *Drosophila *saline. Ring glands of L3 larvae (selected after size: *D. virilis *>3 mm; *D. mojavensis*, *D. sechellia *>2 mm; *D. pseudoobscura *>2.5 mm) were punched out using pulled glass capillaries and spotted directly onto the MALDI target and left to dry. For isolation of the thoracic and abdominal neurohemal sites, the thoracic or abdominal part of the ventral ganglion of adults was cut out and the lateral parts were removed using fine scissors. The dorsal neural sheath was then isolated and freed from cells using tungsten micro-needles. The isolated sheaths were transferred to the MALDI target using pulled glass capillaries and left to dry. This method results in clean spectra from the neurohemal endings [35,36]. For direct profiling of the peritracheal cells, the main branches of the trachea from L3 larvae were dissected free from other tissue and transferred directly onto the MALDI target using fine insect needles. The peritracheal cells were targeted by directing the laser beam to the obtuse angle between the main trachea and the diverging first order trachea.

To remove salts, a small droplet of ice-cold water was added onto the dried tissues, and aspirated off after about 1 s. Small nanoliter volumes of matrix (saturated solution of re-crystallized α-cyano-4-hydroxycinnamic acid in 30% MeOH/30% EtOH/0.1% trifluoroacetic acid for neurohemal organs, or 60% acetonitrile/0.1% trifluoroacetic acid for peritracheal cells were added to the samples using a manual oocyte injector (Drummond Scientific, Broomall, PA, USA). In prior tests MeOH/EtOH/H_2_O (30:30:40) resulted in improved mass spectra compared to 60% MeOH as previously described for *D. melanogaster *[35,36].

### MALDI-TOF mass spectrometry

MALDI-TOF mass spectra were acquired in positive ion mode on a Voyager DE RP mass spectrometer (Applied Biosystems, Darmstadt, Germany) equipped with a pulsed nitrogen laser emitting at 337 nm. Samples were analyzed in reflectron mode using a delayed extraction time of 400 nsec and an accelerating voltage of 20 kV. To suppress matrix signals, the low mass gate was set to 850 Da. Laser power was adjusted to provide optimal signal-to-noise ratios. Data were analyzed using Data Explorer 4.0 software (Applied Biosystems), with a mass tolerance of 0.5 Da.

## Abbreviations

AKH, adipokinetic hormone; AST, allatostatin; CCAP, crustacean cardioactive peptide; CPPB, CAPA precursor peptide B; DAR, *Drosophila *allatostatin receptor; DTK, *Drosophila *tachykinin; ETH, ecdysis-triggering hormone; HUG, hugin; JTT, Jones-Thornton-Taylor; MALDI-TOF, matrix-assisted laser desorption ionization-time of flight; MIP, myoinhibiting peptide; MS, myosuppressin; NPF, neuropeptide F; NPLP, neuropeptide-like precursor; SK, sulfakinin; sNPF, short neuropeptide F.

## Authors' contributions

AG and CW carried out the databank searches and direct peptide profiling, analyzed the mass data, and drafted the manuscript. AG reared the flies. CW carried out sequence alignments and calculated the distances. Both authors read and approved the final manuscript.

## Additional data files

The following additional data are available. Additional data file 1 is a Fasta list containing the prepropeptide sequences as identified by BLAST searches in the 12 *Drosophila *genomes. Additional data file 2 is a Fasta list of the peptide sequences predicted from the identified prepropeptides. Additional data file 3 is a figure showing the frequency of occurrence of peptides in mass spectrometric profiles of the ring gland. Additional data file 4 is a figure containing mass spectrograms on the processing of FMRFa-5^ext ^from the FMRFa-prepropeptide. Additional data file 5 is a table listing the overall average amino acid distances D_so_.

## Supplementary Material

Additional data file 1 Prepropeptide sequences as identified by BLAST searches in the 12 *Drosophila *genomes. Click here for file

Additional data file 2 Peptide sequences predicted from the identified prepropeptides. Click here for file

Additional data file 3 Frequency of occurrence of peptides in mass spectrometric profiles of the ring gland. Click here for file

Additional data file 4 Mass spectrograms on the processing of FMRFa-5^ext ^from the FMRFa-prepropeptide. Click here for file

Additional data file 5 Overall average amino acid distances D_so_. Click here for file
