# Supplementary material for: Molecular evolution of neuropeptides in the genus Drosophila
Source: Genome Biol. 2008 Aug 21;9(8):R131. doi: 10.1186/gb-2008-9-8-r131 (PMC2575521; doi:10.1186/gb-2008-9-8-r131)
Supplement: Additional data file 1 — Prepropeptide sequences as identified by BLAST searches in the 12 Drosophila genomes. [file gb-2008-9-8-r131-S1.doc]

**Prepropeptide sequences of the 12 sequenced *Drosophila* species**

**Adipokinetic hormone**

>Dana AKH dana_GLEANR_10509

MNPKSEVLVAAVLLLLLACVQCQLTFSPDWGKRSVGGASGGSGSFFEPSQGNCKTSNEMLLEIFRFVQSQAQLFLDCKHRE

>Dere AKH dere_GLEANR_15294

MNPKSEVLVAAVLFLLLACVQCQLTFSPDWGKRSVGGAGPGTFFEPQQGSCKTSNEMLLEIFRFVQSQAQLFLDCKHRE

>Dgri AKH dgri_GLEANR_15664

MNPKSEVLIAAVLFFLLACVECQLTFSPNWGKRSVDGGAGGVGGGSGAGGFFESQQGNCKTSNEMLLEIFRFVQSQAQLFLDCKHRE

>Dmel AKH CG1171

MNPKSEVLIAAVLFMLLACVQCQLTFSPDWGKRSVGGAGPGTFFETQQGNCKTSNEMLLEIFRFVQSQAQLFLDCKHRE

>Dmoj AKH dmoj_GLEANR_17339

MNCKTEVLIVAVLFFLLACVECQLTFSPDWGKRSVGGAGGTGGFFEPQQGNCKTSNEMLLEIFRFVQSQAQLFLDCKHRE

>Dper AKH dper_GLEANR_12960

MNTKSEVIIIAAVLCFLLACVEGQLTFSPDWGKRSVGGAGGGSSGVFFEPQQGNCKTSNEMLLEIFRFVQSQAQLFLDCKHRE

>Dpse AKH dpse_GLEANR_8683

MNTKSEVIIIAAVLCFLLACVEGQLTFSPDWGKRSVGGAGGGSSGVFFEPQQGNCKTSNEMLLEIFRFVQSQAQLFLDCKHRE

>Dsec AKH dsec_GLEANR_15385

MNPKSEVLIAAVLFLLLACVQCQLTFSPDWGKRSVGGAGPGTFFEPQQGNCKTSNEMLLEIFRFVQTQAQLFLDCKHRE

>Dsim AKH dsim_GLEANR_13842

MNPKSEVLIAAVLFLLLACVQCQLTFSPDWGKRSVGGAGPGTFFEPQQGNCKTSNEMLLEIFRFVQSQAQLFLDCKHRE

>Dvir AKH dvir_GLEANR_12767

MNPKSEVLIAAVLFFLLACVQCQLTFSPDWGKRSVGGGAGGGGAGGFFEPQQQGNCKTSNEMLLEIFRFVQSQAQLFLDCKHRE

>Dwil AKH dwil_GLEANR_1319

MNTKSQVIIGAVLVVLLAASVECQLTFSPDWGKRSVGGSNSGAGAFFEPSQGNCKTSNEMLLEIFRFVQSQAQLFLDCKHRE

>Dyak AKH dyak_GLEANR_5175

MNPKSEVLIAAVLFLLLACVQCQLTFSPDWGKRSVGGAGPGSFYEPPPANCKTSNEMLLEIFRFVQSQAQLFVDCRHRE

**Allatostatin A**

>Dana ASTa dana_GLEANR_7848

MNSLHAHILLLAVCCVGYIACSPVIGQDQRSPASGDADAEVMLAADEMADNNGDNIDKRMERYAFGLGRRAYMYTNGGAGMKRLPVYNFGLGKRSRPYSFGLGKRSDYDYEQDNEVAYRVPPANYLAAERGVRPGRQNKRTTRPQPFNFGLGRR

>Dere ASTa dere_GLEANR_12357

MNSLHAHILLLAVCCVGYIASSPVIGQDQRSGDTDADVLLAADEMADGGDNIDKRVERYAFGLGRRAYMYTNGGPGMKRLPVYNFGLGKRSRPYSFGLGKRSDYDYEQDNEIDYRVPPANYLAAERAGVRPGRQNKRTTRPQPFNFGLGRR

>Dgri ASTa dgri_GLEANR_3446

MNTMLHAQLLLLALCCLGYVCCSPVIGQESRSQGTNDGDGDGDLLLSAAEDMPDNGGDIDKRMERYAFGLGRRAYMYSNGGAGMKRLPVYNFGLGKRSRPYSFGLGKRSDYDYDQENEIDYRVLPPGYLSVAAGRGQGRQNKRTTRPQPFNFGLGRR

>Dmel ASTa CG13633

MNSLHAHLLLLAVCCVGYIASSPVIGQDQRSGDSDADVLLAADEMADNGGDNIDKRVERYAFGLGRRAYMYTNGGPGMKRLPVYNFGLGKRSRPYSFGLGKRSDYDYDQDNEIDYRVPPANYLAAERAVRPGRQNKRTTRPQPFNFGLGRR

>Dmoj ASTa dmoj_GLEANR_8715

MNMLHTQLLFLALCCLGYVCCTPVIAQDEARNHVSSVNDGDNDMLLNAGEDVQEAGGDIDKRMERYAFGLGRRAYMYNNGGPGMKRLPVYNFGLGKRSRPYSFGLGKRSDYEDEEDNEIDYRALPPGFMAALGESRRQNKRTTRPQPFNFGLGRR

>Dper ASTa dper_GLEANR_14329

MNTLNAHLLLLALCCVGYIACSPVIGQEQRSPVSGEGDADGLLGADEMADNGADIDKRVERYAFGLGRRAYMYNNGGPGMKRLPVYNFGLGKRSRPYSFGLGKRSDYDYEQDNEIDYRVPPANYMGVERAVRPGRQNKRTTRPQPFNFGLGRR

>Dpse ASTa dpse_GLEANR_4872

MNTLNAHLLLLALCCVGYIACSPVIGQEQRSPVSGEGDADGLLGADEMADNGADIDKRVERYAFGLGRRAYMYNNGGPGMKRLPVYNFGLGKRSRPYSFGLGKRSDYDYEQDNEIDYRVPPANYMGVERAVRPGRQNKRTTRPQPFNFGLGRR

>Dsec ASTa dsec_GLEANR_6507

MNSLHAHLLLLAVCCVGYIASSPVIGQDQRSGDSDADVLLAADEMTDNGGDNIDKRVERYAFGLGRRAYMYSNGGPGMKRLPVYNFGLGKRSRPYSFGLGKRSDYDYDQDNEIDYRVPPANYLAAERAVRPGRQNKRTTRPQPFNFGLGRR

>Dsim ASTa dsim_GLEANR_2102

MNSLHAHLLLLAVCCVGYIASSPVIGQDQRSGDSDADVLLAADEMADNGGDNIDKRVERYAFGLGRRAYMYTNGGPGMKRLPVYNFGLGKRSRPYSFGLGKRSDYDYDQDNEIDYRVPPANYLAAERAVRPGRQNKRTTRPQPFNFGLGRR

>Dvir ASTa dvir_GLEANR_8904

MNIVHAQLLLLALYCLGYVCCSPAIGHEARNQVTSDGDGEGDNDLLLSAGEDAPDNGGDIDKRVERYAFGLGRRAYMYTNGGAGMKRLPVYNFGLGKRSRPYSFGLGKRSDYDYDQENEIDYRALPPGFMAAAGRASRQNKRTTRPQPFNFGLGRR

>Dwil ASTa dwil_GLEANR_13569

MNTLHAHTLLLLAICCLGYISCSPVIGQEPRNPVASDGDAENDAVDALIGTAIDDGGDIDKRVERYAFGLGRRAYMYTNAGPGMKRLPVYNFGLGKRSRPYSFGLGKRNDYDYDPDSEMDFRMAPANYIPAERAARPGRQSKRTTRPQPFNFGLGRR

>Dyak ASTa dyak_GLEANR_10675

MNSLHAHILLLAVCCVGYIASSPVIGQDQRSGDSDADVLLAADELADGGDNIDKRVERYAFGLGRRAYMYTNGGPGMKRLPVYNFGLGKRSRPYSFGLGKRGDYDYEQDNEIDYRVPPANYLAAERAVRPGRQNKRTTRPQPFNFGLGRR

**Allatostatin C**

>Dana ASTc dana_GLEANR_14994

MDGQEAEDVRGAYGGGYDMSAQAIYPNIPMDRLQMLYAQYRPTYSAYLRTPTYGNMNELYRLPESKRQVRYRQCYFNPISCFRK

>Dere ASTc dere_GLEANR_10266

MKFVQILLCYGLLLTLLFALSEARPSGAEAGPDSDGLDGQEAEDVRGAYGGGYDMPAQAIYPNIPMDRLQMLFAQYRPTSYSAYLRSPTYGNVNELYRLPESKRQVRYRQCYFNPISCFRK

>Dgri ASTc >dgri_GLEANR_11327

MMKSMQILLCYGLLLTMFFALNEARPSTGEAAADTDGMETPDAEDVRGAFSGYELPAQAIYPNIPMDRLQMLFAQYRPTYSGYLRTPSYANMNELYRLPESKRQVRYRQCYFNPISCFRK

>Dmel ASTc CG149199

MMKFVQILLCYGLLLTLFFALSEARPSGAETGPDSDGLDGQDAEDVRGAYGGGYDMPAQAIYPNIPMDRLQMLFAQYRPTSYSAYLRSPTYGNVNELYRLPESKRQVRYRQCYFNPISCFRK

>Dmoj ASTc dmoj_GLEANR_1740

MVKFGQILLCYGLLLSVFFAVNEARPSAAEATAETDGLDQQDAEDVRAAYGGYEMPAQAIYPNLPMDRLQMLFAQYRPTYSAYLRTPSYGNMNELFRMPESKRQVRYRQCYFNPISCFRK

>Dper ASTc dper_GLEANR_20740

MMKYVQILLCYGLLLALVFSVSEARPSTAETGPDADGLEGQEGEDVRGAYGGGYDMPAQAIYPNIPMDRLQMLFAQYRPTAYLRTPSYGNMNELYRLPESKRQVRYRQCYFNPISCFRK

>Dpse ASTc >dpse_GLEANR_1878

MMKYVQILLCYGLLLALVFSVSEARPSTAETGPDADGLEGQEGEDVRGAYGGGYDMPAQAIYPNIPMDRLQMLFAQYRPTAYLRTPSYGNMNELYRLPESKRQVRYRQCYFNPISCFRK

>Dsec ASTc dsec_GLEANR_1140

MKFVQILLCYGLLLTLLFALSEARPSGADTGPVSNILAERVANVAYSIGFKDSDGLDGQEAEDVRGAYGGGYDMPAQAIYPNIPMDRLQMLFAQYRPTSYSAYLRNPTYGNVNELYRLPESKRQVRYRQCYFNPISCFRK

>Dsim ASTc dsim_GLEANR_5956

MMKFVQILLCYGLLLALLFALSEARPSGAETGPDSDGLDGQEAEDVRGAYGGGYDMPAQAIYPNIPMDRLQMLFAQYRPTSYSAYLRSPTYGNVNELYRLPESKRQVRYRQCYFNPISCFRK

>Dvir ASTc dvir_GLEANR_1786

MMKFVQILLCYGLLLTMFFALNEARPSTAEAGADTDALDTPDVEDVRAAYAGYEMPAQAIYPNIPMDRLQMLFAQYRPTAYLRTPTYGNMNELYRSPESKRQVRYRQCYFNPISCFRK

>Dwil ASTc dwil_GLEANR_18823

MKFVQILLCYGLLLTLLFALSEARPSTGETGPETDGLDGQDSDDVRGSYGGYDMPAQAIYPNIPMDRLQMLFAQYRPSYSAYLRTPTYGNVNELYRLPESKRQVRYRQCYFNPISCFKK

>Dyak ASTc dyak_GLEANR_1330

MKFVQILMCYGLLLTLLFALSEARPSGGETGPDSDVLDGQEAEDVRGAYGGAYDMPAQAIYPNIPMDRLQMLFAQYRPTSYSAYLRSPTYGNVNELYRLPESKRQVRYRQCYFNPISCFRK

**capability (CAPA-periviscerokinins, CAPA-pyrokinin)**

>Dana capa dana_GLEANR_17494

MKFILSMSKLVSAVLVFVLLAEFSAAEMEHDKNRRGANLGLYAFPRVGRGDPSFGNSLRDGLDAGPLDGIYPDASQEDYNDSEFQKRASGLVAFPRVGRGDAELRKFAHLLALQQVLDKRTGPSASSGLWFGPRLGKRSVDSKSFAKGQKEFN

>Dere capa dere_GLEANR_12042

MKSMLVHIVLVIFIIAEFSTAAPLNEKGKDSDHDKNRRGANMGLYAFPRVGRSDPSLANSLRDGLEAGFLDGIYGDASQEDYNEADFQKRASGLVAFPRVGRGDAELRKWAHLLALQQVLDKRTGPSASSGLWFGPRLGKRSVDAQSFADSSKGQKELN

>Dgri capa dgri_GLEANR_1648

MKSFLGLINIVYTILLLVLLTKFSAAEQEQVKHNKNRRGANLGLYAFPRVGRSDPSLVNSLHDANDAVAYDSIYGIGDGSAEDFEEYQKRSSFVPFPRVGRSDSDLRDWAHFLALQQALDKRTGPSASSGLWFGPRLGKRSVNAKDYPSADKEHKDLF

>Dmel capa CG15520

MKSMLVHIVLVIFIIAEFSTAETDHDKNRRGANMGLYAFPRVGRSDPSLANSLRDGLEAGVLDGIYGDASQEDYNEADFQKKASGLVAFPRVGRGDAELRKWAHLLALQQVLDKRTGPSASSGLWFGPRLGKRSVDAKSFADISKGQKELN

>Dmoj capa dmoj_GLEANR_10376

MKSFLGLVNIAYTILLFVLLTKFTAAEVEHDKIRRGANMGLYAFPRVGRSDPSLVNSLHDASDAVTYENIYGMGDASAEDFEDYQKRPGLVAFPRMGRSESELRKWAHLLALQQALDKRTGPSASSGLWFGPRLGKRSINAKDYPAAGKGQKEMY

>Dper capa dper_GLEANR_13968

MKAIFSLYNIVSAILLLVLLAEFSTAELNHDKNRRGANMGLYAFPRVGRSDPSLANSLRDASDAAVFDGLYGDASQEDYNEADYQKRAGLVAFPRVGRSDAELRKFAHLLALQQVLDKRTGPSASSGLWFGPRLGKRSVDAKAFSDASKGQQEFN

>Dpse capa dpse_GLEANR_4672

MKAIFSLYNIVSAILLLVLLAEFSTAELNHDKNRRGANMGLYAFPRVGRSDPSLANSLRDASDAAVFDGLYGDASQEDYNEADYQKRAGLVAFPRVGRSDAELRKFAHLLALQQVLDKRTGPSASSGLWFGPRLGKRSVDAKAFSDASKGQQEFN

>Dsec capa dsec_GLEANR_12636

MKSMLVHIVLVIFIIAEFSTAAPLKEKGKETDHDKNRRGANMGLYAFPRVGRSDPSLANSLRDGLEAGVLDGIYGDASQEDYNEADFQKRASGLVAFPRVGRGDAELRKWAHLLALQQVLDKRTGPSASSGLWFGPRLGKRSVDAKSFTDSSKGQKELN

>Dsim capa dsim_GLEANR_1772

MKSMLVHIVLVIFIIAEFSTAETDHDKNRRGANMGLYAFPRVGRSDPSLANSLRDGLEAGVLDGIYGDASQEDYNEADFQKRASGLVAFPRVGRGDAELRKWAHLLALQQVLDKRTGPSASSGLWFGPRLGKRSVDAKSFADSSKGQKELN

>Dvir capa dvir_GLEANR_10573

MKSFLGLLNIAYTILLLVLLTKFSAAVEHDKIRRGANMGLYTFPRVGRSDPSLVNSLHDDNDAVAYETLYGFGDASAEDFEEYQKRASLVPFPRVGRSDSELRKWAHLLALQQALDKRTGPSASSGMWFGPRLGKRSINVKDYPADTKGQKELF

>Dwil capa dwil_GLEANR_14413

MKAVLGLDNVAYAILVFVVLAKFSEAELDHDKNRRGANMGLYAFPRVGRSDPSLANGVHDAGESSAFESTYGDSSQEDYEDYQKRASLVPFPRVGRQDPELRKLARLVARQQAYDKRTGPNASSGLWFGPRLGKRSVDAKDYPVAADKGQKEFN

>Dyak capa dyak_GLEANR_10340

MKSMLVHIVLVIFIIAEFSTAASLKEKETDHDKNRRGANMGLYAFPRVGRSDPSLANSLRDGLEAGVLDGIYGDASQEDYNEADFQKRASGLVAFPRVGRGDAELRKWAHLLALQQVLDKRTGPSASSGLWFGPRLGKRSVDAQSFADSSKGQKELN

**Crustacean cardioactive peptide**

>Dana CCAP dana_GLEANR_17962

MTSPIKNSLRMAVLLVFVISCQASLERENSEVNNLSNHKLSGVIQWKYEKRPFCNAFTGCGRKRTSYPSYPPFSLIKRNEIEEKPYNNEYLSEGLSDLIDINAEPAVENVQKQIMSQAKIFEAIKEASKEIFRQKNKQKLLENGQKLQLQEERENI

>Dere CCAP dere_GLEANR_12510

MRTFMRISLRLVALLVCFICSQASLERENEGPNMANHKLSGVIQWKYEKRPFCNAFTGCGRKRTYPSYPPFSLFKRNEIEEKPYNNEYLSEGLSDLIDINAEPAVENVQKQIMSQAKIFEAIKEASKEIFRQKNKQKMLQNEKEMQQLEERESK

>Dgri CCAP dgri_GLEANR_2879

MRTTLEIFLCLAAIFLFAYSADASLETENNEVNHKLSGVIQWKFEKRPFCNAFTGCGRKRTSYPSYPPFSLFKRNEIEEKPYNNEYLSEGLSDLIDINAEPAVENVQKQIMSQAKIFEAIKEASKEIFRQKNKQKLLESQHQQQLQDQQLDQGDNN >Dmel CCAP CG4910

MRISLRLLALLACAICSQASLERENNEGTNMANHKLSGVIQWKYEKRPFCNAFTGCGRKRTYPSYPPFSLFKRNEVEEKPYNNEYLSEGLSDLIDINAEPAVENVQKQIMSQAKIFEAIKEASKEIFRQKNKQKMLQNEKEMQQLEERESK

>Dmoj CCAP dmoj_GLEANR_9507

MSASVEICFYLTAILICMYTANASLETENNEVNHKLSGVIQWKYEKRPFCNAFTGCGRKRTSYPSYPPFSLIKRNELEEKPYNNNEYLSEGLSDLIDINAEPAVENVQKQIMSQAKIFEAIKEASKEIFRQKNKQKMLENEQQLQQQQQEQLLEQRDNN

>Dper CCAP dper_GLEANR_6115

MKSSLKMCLGLVVLLICMLSGNASLERENNEVNNLANHKLSGVIQWKYEKRPFCNAFTGCGRKRTSYPSYPPFSLIKRNELEEKPYNNEYLSEGLSDLIDINAEPAVENVQKQIMSQAKIFEAIKEASKEIFRQKSNRQKMLENGQRLQMQEERENI

>Dpse CCAP dpse_GLEANR_5155

MKSSLKMCLGLVVLLICMLSGNASLERENNEVNNLANHKLSGVIQWKYEKRPFCNAFTGCGRKRTSYPSYPPFSLIKRNELEEKPYNNEYLSEGLSDLIDINAEPAVENVQKQIMSQAKIFEAIKEASKEIFRQKSNRQKMLENGQRLQMQEERENI

>Dsec CCAP dsec_GLEANR_6663

MRTSMRISLRLLALLACAICSQASLERENNEGPNMANHKLSGVIQWKYEKRPFCNAFTGCGRKRTYPSYPPFSLFKRNEVDEKPYNNEYLSEGLSDLIDINAEPAVENVQKQIMSQAKIFEAIKEASKEIFRQKNKQKMLQNEKEMQQLEERESK

>Dsim CCAP dsim_GLEANR_2259

MRTSMRISLRLLALLACAICSQASLERENNEGPNMANHKLSGVIQWKYEKRPFCNAFTGCGRKRTYPSYPPFSLFKRNEVEEKPYNNEYLSEGLSDLIDINAEPAVENVQKQIMSQAKIFEAIKEASKEIFRQKNKQKMLQNEKEMQQLEERESK

>Dvir CCAP dvir_GLEANR_9647

MRATVEIFLGLAAIIICLYTANASLEIENNEVNHKLSGVIQWKYEKRPFCNAFTGCGRKRTSYPSYPPFSLIKRNEPEEKPYNNEYLSEGLSDLIDINAEPAVENVQKQIMSQAKIFEAIKEASKEIFRQKNKQKMLENEQQQQLEQRENN

>Dwil CCAP dwil_GLEANR_11822

MNISTKLCLWLALLLFSVLCGSASLERERQTNEVNNLSNHKLSGVIQWKYEKRPFCNAFTGCGRKRTSYPSYPPFSLIKRNDIDDKPYNNEYMSEGLSDLIDINAEPAVENVQKQIMSQAKIFEAIKEASKEIFRQKNKQKLLESQQQQQQQQLQQQQQMQEQERENI

>Dyak CCAP dyak_GLEANR_7751

MRISLRLIALLACFICSQASLERENNEGPNMANHKLSGVIQWKYEKRPFCNAFTGCGRKRTYPSYPPFSLFKRNEVEEKPYNNEYLSEGLSDLIDINAEPAVENVQKQIMSQAKIFEAIKEASKEIFRQKNKQKMLQNEKELQQLEERDSK

**Corazonin**

>Dana Crz dana_GLEANR_17438

MLRLMLLPLFLFTLSMACMGQTFQYSRGWTNGKRALNTGSPLLGNGHLHRSNELSFSDLYDLQDWSSDRRLERCLTQLQRSLLSRNCVPGVELNLNRMDTDSGDTNLHSRPNNNENENLVYSNPKQNRRQSNELLEELSVAAVGGSSEPNVFGKH

>Dere Crz dere_GLEANR_1787

MLRLLLLPLFLFTLSMCMGQTFQYSRGWTNGKRSFNAASPLLTTGHLHRGSELGLSDLYDLQEWTSDRRLERCLSQLQRSLIARNCVPGSDFNANRVDPDPESSAHPRLGNINNENVLYSSANVPNRHRQSNELLEELSAAGGASAEPNVFGKH

>Dgri crz dgri_GLEANR_306

MLRVLLLPLLLFTLSMACMGQTFQYSRGWTNGKRAPPAALLSNGHNLGLLDLYDIQDKPTDLKLERCLMQLQHFVGNAMLHRSFANALAYNANRPETEAETRGLNLHPLHPSANNNNNNENSLFPNSNNHQSNEIFEALAASAPGVDSVEPNDYGKH

>Dmel Crz CG3302

MLRLLLLPLFLFTLSMCMGQTFQYSRGWTNGKRSFNAASPLLANGHLHRASELGLTDLYDLQDWSSDRRLERCLSQLQRSLIARNCVPGSDFNANRVDPDPENSAHPRLSNSNGENVLYSSANIPNRHRQSNELLEELSAAGGASAEPNVFGKH

>Dmoj Crz dmoj_GLEANR_10713

MLRLLLLPLFLFTLSMVCMGQTFQYSRGWTNGKRATPSALLNTGHNLGLLDAFEMQEKPSDIKLERCLMQLQHLAGNALLHHSFANGLAYNGNRPDPTDLNIHARPRPSANNNDNNLLYPRDNQHQSNELYETVADPVEPVDFGKH

>Dper Crz dper_GLEANR_5617

MMRLLLLPLFLFTLSMACMGQTFQYSRGWTNGKRALTPPSLLSHGHFNRASDLGFSDLYDVQDWSSERRLERCLAQLQRSLLSRVYGSVVDFNANRPEPDSSDSGSSRNRANNNNENVLYPTPIQNRHHSSNELLEEISAAVAGSGPTGAGSGEPSVFGKH

>Dpse Crz dpse_GLEANR_5517

MMRLLLLPLFLFTLSMACMGQTFQYSRGWTNGKRALTPPSLLSHGHFNRASDLGFSDLYDVQDWSSERRLERCLAQLQRSLLSRVYGSVVDFNANRPEPDSSDSGSSRNRANNNNENVLYPTPIQNRHHSSNELLEEISAAVAGSGPTGAGSGEPSVFGKH

>Dsec Crz dsec_GLEANR_7166

MLRLLLLPLFLFTLSMCMGQTFQYSRGWTNGKRSFNAASPLLANGHLHRGSELGLTDLYDLKDWSSDRRLERNCVPGSDFNTNLVDPDPENSVHPRLSNINGENVLYSSANIPNRHRQSNELLEELSAAGGASAEPNVFGKH

>Dsim Crz dsim_GLEANR_2760

MLRLLLLPLFLFTLSMCMGQTFQYSRGWTNGKRSFNAASPLLANGHLHRGSELGLTDLYDLQDWSSDRRLERCLSQLQRSLIARNCVPGSDFNANRVDPDPENSVHPRLSNINGENVLYSSANIPNRHRQSNELLEELSAAGGASAEPNVFGKH

>Dvir Crz dvir_GLEANR_14362

MLRLLLLPLFLFTLSMACMGQTFQYSRGWTNGKRAPPAALVTNGHNLGLLDIYDIQDRPTDIKLERCLLQLQHFVGNALLHRSFANGLAYSASRPDPETDVRSINIHSRPGSGNNNIENSLYPNVNHRQSNELFEALNAPGPDAVEPNDYGKH

>Dwil Crz dwil_GLEANR_11565

MLRLLLLPLFLFTLSMACMTHGQTFQYSRGWTNGKRSSLSSTPTLLNNGQHFHRSNDVSFADFYDFPATISERRLERCLTQLQHFVGNSLLHRSFATGLDTDSSDAQSSSNENSPYTNPKRHHQSSELFEDLNAAVGSVPAAGPGEPNDFIKH

>Dyak Crz dyak_GLEANR_7945

MLRLLLLPLFLFTLSMCMGQTFQYSRGWTNGKRSFNAASPLLTTGHLHRSSELGLSDLYDLQDWSGERRLERCLSQLQRSLIARNCVPGSDFNANRVDPDPESSAHPRLGSINNENALYSSANVPNRHRQSNELLEELSAAGGASAEPNVFGKH

**Diuretic hormone 31**

>Dana DH31 dana_GLEANR_15132

MPTIIPNVQNTVERLWTKQTLIFHFDDSHNSQSNGGGYGGGYNELEEVPDDLLMELMTRFGRTIIRARNDLENSKRTVDFGLARGYSGTQEAKHRMGLAAANFPGGPGRRRRSETDV

>Dere DH31 dere_GLEANR_8831

MTNRCACFALAFLLFCLLAISSIEAAPMPSQSNGGYGGAGYNELEEVPDDLLMELMTRFGRTIIRARNDLENSKRTVDFGLARGYSGTQEAKHRMGLAAANFAGGPGRRRRSETDV

>Dgri DH31 dgri_GLEANR_10692

MTNRFAFCALALLIICLLAISRTDAAPMPSQSSNNGYGGYNELEEVPDDLLMELMTRFGRTIIRARNDLENSKRTVDFGLARGYSGTQEAKHRMGLAAANFPGGPGRRRRSETDA

>Dmel DH31 CG13094

MTNRCACFALAFLLFCLLAISSIEAAPMPSQSNGGYGGAGYNELEEVPDDLLMELMTRFGRTIIRARNDLENSKRTVDFGLARGYSGTQEAKHRMGLAAANFAGGPGRRRRSETDV

>Dmoj DH31 dmoj_GLEANR_2431

MTNRIAFAALALMSICLLAISRTDAAPMPSQSSNGGYGGYNELEEVPDDLLMELMTRFGRTIIRARNDLENSKRTVDFGLARGYSGTQEAKHRMGLAAANFPGGPGRRRRSETDA

>Dper DH31 dper_GLEANR_8219

MTNRFACFAVALLAVCLLAISSTEAAPMPSQSNGGGGYGGGAGYNELEEVPDDLLMELMTRFGRTIIRARNDLENSKRTVDFGLARGYSGTQEAKHRMGLAAANFAGGPGRRRRSETDV

>Dpse DH31 dpse_GLEANR_16889

MTNRFACFAVALLAVCLLAISSTEAAPMPSQSNGGGGYGGGAGYNELEEVPDDLLMELMTRFGRTIIRARNDLENSKRTVDFGLARGYSGTQEAKHRMGLAAANFAGGPGRRRRSETDV

>Dsec DH31 dsec_GLEANR_1325

MTNRCACFALAFLLFCLLAISSIEAAPMPSQSNGGYGGAGYNELEEVPDDLLMELMTRFGRTIIRARNDLENSKRTVDFGLARGYSGTQEAKHRMGLAAANFAGGPGRRRRSETDV

>Dsim DH31 dsim_GLEANR_6148

MTNRCACFALAFLLFCLLAISSIEAAPMPSQSNGGYGGAGYNELEEVPDDLLMELMTRFGRTIIRARNDLENSKRTVDFGLARGYSGTQEAKHRMGLAAANFAGGPGRRRRSETDV

>Dvir DH31 dvir_GLEANR_2763

MTNRFACCTLALMTIYLLAISRTDAAPMPRQSANGGYGAYNELEEVPDDLLMELMTRFGRTIIRARNDLENSKRTVDFGLARGYSGTQEAKHRMGLAAANFPGGPGRRRRSETDA

>Dwil DH31 dwil_GLEANR_8975

MTNRFAFTALTFLLMCLLAISSNEAAPMPSQSNGGGGGYGGGYGGNELEEVPDDLLMELMTRFGRTIIRARNDLENSKRTVDFGLARGYSGTQEAKHRMGLAAANFPGGPGRRRRSETDV

>Dyak DH31 dyak_GLEANR_1082

MTNRCACFALAFLLFCLLAISSIEAAPMPSQSNGGYGGAGYNELEEVPDDLLMELMTRFGRTIIRARNDLENSKRTVDFGLARGYSGTQEAKHRMGLAAANFAGGPGRRRRSETDV

**Diuretic hormone 44 (CRF-related diuretic hormone)**

>Dana DH44 dana_GLEANR_17879

MMKATAWFCPVLLTLLCATRLVCTAQRGAGSGTGTGVGGTAAGAGPEAGGSGRTNGYPLDYPDARNIQDDFLLAKRNKPSLSIVNPLDVLRQRLLLEIARRQMKENSRQVELNRAILKNVGKRFMIRGGGAPKVPTRRYREREREWEREREPEELQQLEELELEQEQQQELVREQEEQALRQQLLPWKHFPSQLWSYGWTHSQSPYSALQLADSLQQKTTRPQSLPGQKLPSYAKKSLNVAGLGLGVRAVRGHRAANGNEAANETTNHDNDENENGYGSSKNAAGYVDDEDDDGEAGFDLFEEVVPSAPKLLNPLYHDL

>Dere DH44 dere_GLEANR_192

MMKATAWFCPVLLTLLCATRLVCTAQRGAAAAGGAAGGSGAAAGGAELGGSGRTNGYPLDYPDGTRNTQDDFLLAKRNKPSLSIVNPLDVLRQRLLLEIARRQMKENSRQVELNRAILKNVGKRFMLRGGGVGGVGGSGAGGLAPKVSRRYRQQWPLERELEQERQKDAVPEEQLYRQQLLPWKHFPSQLWSYGWTQSPYKESSQFVDSQQSASTGPQSQALPKQLQLQLLSYAKKPLDVAGMGLARHRVSGNEANETNHENDDGNGASKNTARYVDDDDEGEDSYNDLGAEGVGQGLRLGMGLGLGLERFEVMEDKPNWANEEPNEVMMVNANERVPWSFPYRFHKSQHNVN

>Dgri DH44 dgri_GLEANR_2663

MMKATAWFCPVLLTLLSATRLVCTAQRGAAATTGGAEGGGAAAAAAVAAAAAALAAGSAGYANGYPLDYLDAQTIQDEYSLVKRNKPSLSIVNPLDVLRQRLLLEIARRQMKENTRQVELNRAILKNVGKRVFLEPATWGNTHYQQHQQQLERQQEAWREQLRREQLQQQHFPSQLWRFGWPQSTTGTSTGTSSVSGSGSGSGTPSASSHIFDFLQQPATRPRQQTAAQQLLDDTNKSLNTLGTLGLGVAKHPQQQQQQPEQTNGNEIANETNHENGHRKSLPVGKSATGVVNDEDADPDEEQDQDDLLDDLNLNWGNEQSDDLESVPEAANANGQLPWHLIYRVHKNPHYAN

>Dmel DH44 CG8348

MMKATAWFCPVLLTLLCATRLVCTAQRGAVGAGGAAGGSGAAAGGAEVGGSGRTNGYPLDYPDGTRNSQDDFLLAKRNKPSLSIVNPLDVLRQRLLLEIARRQMKENSRQVELNRAILKNVGKRVVLRGGGGGGGSGAGGLAPKVSRRYRQQWPVERELERERQRERERERDAVREEQLDRQQLLPWKHFPSQLWSYGWALSPYKESSQLQFADSQQSASTGPQSQALPKQLQLLSYAKKPLDVAGMSLARHRVSGNEANETNHENDDGNGASKNPARYVDDGDNEGEDSYNDVGTEGVGLGLGMGVGLGLERFEVLEDKPNWANEEPNELVVVNANDRVPWSFPYRFHKSQHNVN

>Dmoj DH44 dmoj_GLEANR_9254

MMKATAWFCPVLLTLLSATRLVCTAQRGAPATAAGAAGGGSANGFPLDYLDVQAVQDEYSLAKRNKPSLSIVNPLDVLRQRLLLEIARRQMKENTRQVELNRAILKNVGKRVFLEPAPWGSMRHKLLQQQQQQQQVQLEREREREQLRREQLQQQHFPSQLWGYSGNPAGSSDPSTASAASRFFDFLHRPAKEQQQQLLLDKSLKEGVAKHHQSNGNEIANDTNHENGHRKSATDLMSTEDELDNSGEDRDEMLYEPNMNWASEQHMDDMDRSLEAANGNSRMPWRLIYRMHKASHYAN

>Dper DH44 dper_GLEANR_12542

MMKATAWFCPVLLTLLCATRLVCTAQRGTGTGTDAEQQPAGNSHTNGYPLDYLDARNTQDDFLLAKRNKPSLSIVNPLDVLRQRLLLEIARRQMKENTRQVELNRAILKNVGKRVYLRPAAAEAMLSSPYSQQLERALARSNKRQQKMHWTNFPSPLQWSYAMPQSPDTSQFQSQSQKSQARKLFNYAKKSLNGAGAGPGLGMSVAAKHPADGNEVANEKYHENHGDRNAAMASKNATIYVADTDEVDDDNENENEEEESRVEQAFEEDKPNLEPHGMAAGVDNTIEYIPWNLLYRYLPQRKHYNDI

>Dpse DH44 dpse_GLEANR_6232

MMKATAWFCPVLLTLLCATRLVCTAQRGTGTGTGAEQQPAGNSHTNGYPLDYLDARNTQDDFLLTKRNKPSLSIVNPLDVLRQRLLLEIARRQMKENTRQVELNRAILKNVGKRVYLRPAAAEAILSRPYSQQLERALARSNKRQQKMHWTNFPSPLQWSYAMPQSPDTSQFQSPSQSQKSQAQKLFNYAKKSLNGPGLGMSVAAKHPADGNEVANEKYHENHGDRNAAMASKNATIYVADTDEVDDENGNDNDNEEEESRVEQAFEEDKPNLEPHGMAAGVDNTIEYIPWNLLYHYLPQRKHYNDI

>Dsec DH44 dsec_GLEANR_6848

MMKATAWFCPVLLTLLCATRLVCTAQRGAVGAGGAAGGSGAAAGAAEVGGSGRTNGYPLDYPDGIRNSQDDFLLAKRNKPSLSIVNPLDVLRQRLLLEIARRQMKENSRQVELNRAILKNVGKRVMLRGGGGGSGAGGLAPKVSRRYRQQWPVERELERERQRERERDAVREEQLDRQQLLPWKHFPSQLWSYGWAQSPYKESSQLQFADSQQSASTGPQSQALQKQLQLLSYAKKPLDVAGMGLARHRVSGNEANETNHENDDGNGASKNPARYVDDDDDEGEDSYNDVGTEGVGLGLGMGVGLGLERFEVLEDKPNWANEEPNELMVVNANDRVPWSFPYRFHKSQHNVN

>Dsim DH44 dsim_GLEANR_2453

MMKATAWFCPVLLTLLCATRLVCTAQRGAAGAGGAAGGSGAAAGGAEVGGSGRTNGYPLDYPDGIRNSQDDFLLAKRNKPSLSIVNPLDVLRQRLLLEIARRQMKENSRQVELNRAILKNVGKRVMLRGGGGGGGSGAGGLAPKVSRRYRQQWPVERELERERQRERERDAVREEQLDRQQLLPWKHFPSQLWSYGWAQSPYKESSQLQFADSQQSASTGPQSQALPKQLQLLSYAKKPLDVAGMGLARHRVSGNEANETNHENDDGNGASKNAARYVDDDDDEGEDSYNDVGTEGVGLGLGMGVGLGLERFEVLEDKPNWANEEPNELVVVNANDRVPWSFPYRFHKSQHNVN

>Dvir DH44 dvir_GLEANR_10792

MMKATAWFCPVLLTLLSATRFVCTAQRGAATTAAGGGDAGGADGAGGGSAPAAAAAGGAGYANGYPLDYLDAQAVQAEYSLVKRNKPSLSIVNPLDVLRQRLLLEIARRQMKENTRQVELNRAILKNVGKRVFLEPATWGNSRYQQQQQQQQLERQRELLRREQLQQQHFPSQLWSYGWPQSDSGSVAGSVAGNAATPPVSSSAMAASRFFDFLQPPAKQQQLLDDANKSLNAGALGQGMAKHQQPNGNEIANETNHLNGNRKSATSLLAGMEEDQEDNGEDQDEMLDELNLNWANEQPMDDMDRPLEAANANGRLPWRLIYRMHKNPHYAN

>Dwil DH44 dwil_GLEANR_11342

MMKATAWFCPVILTLLCATRLVCTAQRGGGGGGASGSGNNNNGYPLDYLDDAQAIQDDYLLWAKRNKPSLSIVNPLDVLRQRLLLEIARRQMKENTRQVELNRAILKNVGKRVFVEPRRSYQLDSMREQQLQHFPSQLWSLSQSSEPSLSSPVFPSSSSSLLSPSSSRFFDFLQRAQSQPRPRTITAAEQESEAEATSAQQKSLNVLAKHQSNGNEIANETNHENGHGQSATGNAVANNMDVESQTEQEQEQDRVLAELEDNLNWANEEPNDIADNEDNANVVSRHLPWRLLYGQHKHHHYAN

>Dyak DH44 dyak_GLEANR_9555

MMKATAWFCPVLLTLLCATRLVCTAQRGAVGAGGAGGAAGGSGATAGGAEVGSGRTNGYPLDYPDGTRNTQDDFLLAKRNKPSLSIVNPLDVLRQRLLLEIARRQMKENSRQVELNRAILKNVGKRVRGGGSGAGGLAPKVSRRYRQQWPAVERELERERQGDAVPEEQLDRQQLLPWKHFPSQLWSYGWAQSPVAYKESSQSQFVDSQQSASTGPQSQALPKQLQLQLLSYAKKPLDVAGMGLARHRVSGNEANETNHENDDGNVNGNGNGASKNPARYVVDDDDEGEDSYNDVGPEGVGLGLGALGVGLGLGLERFEVLDDKPNWANEEPNEVMVVNANERVPWSFPYRFHKSQHNVN

**Ecdysis-triggering hormone**

>Dana ETH dana_GLEANR_11589

MRSLTILALLILLPLVAITRADDSPGFFLKITKNVPRLGKRGETRMKNLKTIPRIGRSEQVSLPEKLSVLRLKPALQSGVTPLMAWLWDLDVAPIKRRLPSGEEGGGKEQELNVVQPVNSNTLMELLDNNAIPSEQVKFVHWRDFDRALQTDLDLYAKVIQLGRRPDHRLKQTLSLGSFVPIFGEDGDQNSAFMMYNNNDDRDLYGGGSRYGGNFLKYNHI

>Dere ETH dere_GLEANR_4732

MRFITVLSVSLLVALVAISQADDSSPGFFLKITKNVPRLGKRGENFAIKNLRTIPRIGRSEHSSVTPLLAWLWDLDTSPSKRRLPAGESAAKEQELNVVQPVNSNTLLELLDNNAIPSEQVKFVHWKDFDRALQADADLYNKVIQLGRRPDQHLKQTLSFGSYVPIFGDDQNPDFMMYKNNEDQELYGGGNRYDRHFLKYNTL

>Dgri ETH dgri_GLEANR_5687

MNSATILLFAVLLLAELWCPGQADESPGFFLKITKNVPRLGKRSEGFPMKNMKTIPRIGRSDPKQVSRPHFASNSISKIDVFQASVTSLLAWLWNMDMDLTQPQLSKRRLGNNNGVANVERELNVVQPVNSNTLLELLNRNAIPSEHVKFVHWKDFDRALQADAELYAKVIHLGRDPDQRLKHDLSFSSYVPIFGSDVDGQGNDFMLYNKDDRDLYTSSGRFDSNFMQYNRL

>Dmel ETH CG18105

MRIITVLSVSLLVGLVAISQADDSSPGFFLKITKNVPRLGKRGENFAIKNLKTIPRIGRSEHSSVTPLLAWLWDLETSPSKRRLPAGESPAKEQELNVVQPVNSNTLLELLDNNAIPSEQVKFVHWKDFDRALQADADLYSKVIQLGRRPDQHLKQTLSFGSFVPIFGDEQNPDFMMYKNNEDQELYGGGNRYDRQFLKYNIL

>Dmoj ETH dmoj_GLEANR_4436

MNRLTILFAVLLLAELWIAGYADESPGFFLKITKNVPRLGKRGEAFLMKNMKTIPRIGRSDPEASITPLLAWLWNLDADPQFSKRRLPSSGAAGSVEHELSVVQPVNSNTLFELLDRNAIPSEHVKFVHWKDFDRALQVDTELYTKIIHLGRDPDQRLKQDLSFNSYIPIFGSDDAKSNDFMLYNKDDRDLYGGYDRNFMQYNRL

>Dper ETH dper_GLEANR_19484

MRSLTVLGVILLLVLVATSQADDSPGFFLKITKNVPRLGKRSESFGMKNLKTIPRIGRSEQQTAVTPLLTWLWDVDVSQPSKRRLATGEAAGQERELTVVQPVNANTLMELLDNNAIPSEHVKFVHWKDFDRALQSDTDLYAKVIQLGRRPDHRLKEDLNFNSYVPIFDGNGDQNAPFMMYNNNEDRDLYGGGNRYDRNFLKYNHL

>Dpse ETH dpse_GLEANR_15822

MRSLTVLGVILLLVLVATSQADDSPGFFLKITKNVPRLGKRSESFGMKNLKTIPRIGRSEQQTAVTPLLTWLWDVDVSQPSKRRLATGEAAGQERELTVVQPVNANTLMELLDNNAIPSEHVKFVHWKDFDRALQSDTDLYAKVIQLGRRPDHRLKEDLNFNSYVPIFDGNGDQNAPFMMYNNNDERDLYGGGNRYDRNFLKYNHL

>Dsec ETH dsec_GLEANR_12146

MRIITVLSVSLLVALVAISQADDSSPGFFLKITKNVPRLGKRGENFAIKNLKTIPRIGRSEHSSVTPLLAWLWDLDTSPSKRRLPAGESAAKEQELNVVQPVNSNTLLELLDNNAIPSEQVKFVHWKDFDRALQADADLYSKVIQLGRRPDQHLKQTLSFGSFVPIFGDDHNPDFMMYKNNEDQELYGGGNRYDRQFLKYNIL

>Dsim ETH dsim_GLEANR_8917

MRIITVLSVSLLVALVAISQADDSSPGFFLKITKNVPRLGKRGENFAIKNLKTIPRIGRSEHSSVTPLLAWLWDLDTSPSKRRLPAGESAAKEQELNVVQPVNSNTLLELLDNNAIPSEQVKFVHWKDFDRALQADADLYSKVIQLGRRPDQHLKQTLSFGSFVPIFGDDQNPDFMMYKNNEDQELYGGGNRYGSQFLKYNIL

>Dvir ETH dvir_GLEANR_6466

MSSPTILLYAVLLLAGLWSSAHADESPGFFLKITKNVPRLGKRSEGFPMKNIKTIPRIGRSDPQASVTPLLAWLWDMDMDMAQPQLSKRRLPNNVGAGHVEHELNVVQPVNSNTLLELLDRNAIPSEHVKFVHWKDFDRALQADTELYDKVIHLGRDPDQRLKQDLNFNSYIPIFGSDDGLGNNFMLYNTDDSDLYGSNNRYNRNFMEYNRL

>Dwil ETH dwil_GLEANR_4747

MRLLTILAFVGLVLNLVLADDSPGFFLKITKNVPRLGKRSENFVMKNIPRIGRSSFIGGQQSSSVTPLMAWLWDLDIEPVKRRYPTSDQIKALERELNVVQPVNSNTLIELLDKNAIPSEQVKFVHWKDFDRALQSDMDLYAKVIQLGRRPDQRLKEDLNLNSYIPIFGTNNEQNGDFIMLNSNDRDLYGSRSRFDRNFMKYNHL

>Dyak ETH dyak_GLEANR_11745

MRFITVLSVSLLVAIVAISQADDSSPGFFLKITKNVPRLGKRGENFSLKNLKTIPRIGRSEHSSVTPLLAWLWDLDTSPSKRRLPAGESATKEQELNVVQPVNSNTLLELLDNNAIPSEQVKFVHWKDFDRALQVDADLYSKVIQLGRRPDRHLKQTLSFGSFVPIFGDDQNPDFMMYKNNEDQELYGGGNRYDRNFLKYNTL

**FMRFamide-like peptides**

>Dana FMRF dana_GLEANR_13576

MGIALMFLLALYQMQSAIHSEIIETPFAAGGNSLLESPDVTSEILEQVPPQDNELLEPEKAQLEFNHPISVIGVDYGKNAVILRFQKHGRKPRYKYDPELEAKRRTLQDNFMHFGKRQVEELPLEEPEPELDAVKRSAIPEQDLMRYGRDPKQDFMRFGRDPKQDFMRFGRTPSDFMRFGRAPSDFMRFGRTPLENFMRLGRSDNFMRFGRSPHEEFRSPKQDFMRFGRPDNFMRFGRSAPPEFARNGKMDSNFMRFGKSLMPTTLEHNQTKPAKLTESILKKKQQDQTKSNVDINSADDEQEPFFSHLKSNLLHRSSLLLFVVAINCLTTHPAFQCIASTFRLLDRSLEDYRDQYVGFIENAQSVSEAAKYPKITTHYTVHPREKDERWKEVDMERCIEEVDLVIVGGGPAGMSAAIRAKQLAAEKDQ

>Dere FMRF dere_GLEANR_8878

MGIALMFLLALYQMQSAIHSEIIETPNYGNSLQDTEPEVSTPQDNDLVDALLGNDQTERAELEFRHPISVIGIDYSKNAVVLHFQKHGRKPRYKYDPELEAKRRSVQDNFMHFGKRQAEQLPPEGSYGGSDELEGMAKRAAMDRYGRDPKQDFMRFGRDPKQDFMRFGRDPKQDLMRFGRDPKQDFMRFGRDPKQDFMRFGRDPKQDFMRFGRTPAEDFMRFGRTPAEDFMRFGRSDNFMRFGRSPHEELRSPKQDFMRFGRPDNFMRFGRSAPQDFVRSGKMDSNFIRFGKSVKPEAPESKQTKPIQGNLGERSPVDKAMTELFKKQELQDQQDQQVKSDGQATTTEDGSVEQDQFFGQ

>Dgri FMRF dgri_GLEANR_4515

MGIALMFLLALYQMQSAIYSEIIDTPNYTDNALNELEDSSTEPLPAVDNDMLDAMMMMNEKPDQTELELRLPISNIGFDYGKNSMVLRLQKNARKPQLKYDPDYEQKRKSLRDNFMHFGKRQAEQLPQPTGPGYYEMSKRSAMDRYGREPKQDFMRFGRTPSDFMRFGRAPSDFMRFGRDPKQDFMRFGRDPKQDFMRFGRDPKQDFMRFGRDPKQDFMRFGRDPKQDFMRFGRDPKQDFMRFGRDPKQDFMRFGRDPKQDFMRFGRADDFMRFGRNVNYHEEQRSSKPDFMRFGRPDNFMRFGRSPPTEFERNGKMDSNFMRFGKRSPGDGAAGTESNQTKAQLQQNKITADGGKQEQQQPSDDSNTVDKTITMLFDKHHQEPQQQQQQQQQRQPQEEQQLKSSEQNNLEEASAEQFFEP

>Dmel FMRF CG2346

MGIALMFLLALYQMQSAIHSEIIDTPNYAGNSLQDADSEVSPPQDNDLVDALLGNDQTERAELEFRHPISVIGIDYSKNAVVLHFQKHGRKPRYKYDPELEAKRRSVQDNFMHFGKRQAEQLPPEGSYAESDELEGMAKRAAMDRYGRDPKQDFMRFGRDPKQDFMRFGRDPKQDFMRFGRDPKQDFMRFGRDPKQDFMRFGRTPAEDFMRFGRTPAEDFMRFGRSDNFMRFGRSPHEELRSPKQDFMRFGRPDNFMRFGRSAPQDFVRSGKMDSNFIRFGKSLKPAAPESKPVKSNQGNPGERSPVDKAMTELFKKQELQDQQVKNGAQATTTQDGSVEQDQFFGQ

>Dmoj FMRF dmoj_GLEANR_4354

MGIALMFLLALYQMQSAIHSEIIETPSYIENSLLESEEVNPKAVESDILDGLASDKLDQTELEFRYPISAIGIDYNKNSVVLRFQKHAHKPNFKYDPDYDIKRKSMQDNFMRFGKRQAEQMPQATGPGYYEAAKRSGMDRYGRDPKQDFMRFGRSPSDFMRFGRSPSDFMRFGRDPSQDFMRFGRSDNFMRFGRNMNFHEELRSPKQDFMRFGRPDNFMRFGRAAPSDFERFGKMDSNFMRFGKSVNRSGAGTGAGTGTEGGLASSSESKQLNKLDGKQQGTEETNPVDKAMSMLFNKQQQQGQQGQGQAQRSTHDQQQLKSGEQTESEEQFYEP

>Dper FMRF dper_GLEANR_18605

MGIALMFLLALYQMQSAIHSEIIESPNFGSNALLESDTDADQGSSFQTGHREILQDAEEKPDQTEQEFRYPISAIGIDYARNSVILRFQKHGRRQRLKYDPELEAKRRSLQDNFMHFGKRQEEQMPPDESYGSDGEEGMVKRSDMDRYSRDPKQDFMRFGRDPKQDFMRFGRDPKQDFMRFGRDPSDFMRFGRAPSDFMRFGRTPSDFMRFGRTPSDFMRFGRSDNFMRLGRSPHEELRSPKQDFMRFGRPDNFMRFGRSAPPEFERYGKMDSNFMRFGKSANPAAPPASGAPESNQTKSQPASNQPGERSPVDKAMTILFRKQEQQRQSKSGEQTEHTAEGGSVEQEQFYGQ

>Dpse FMRF dpse_GLEANR_14199

MGIALMFLLALYQMQSAIHSEIIESPNFGSNALLESDTDADQGSSFQTGHREILQDAEEKPDQTELEFRYPISAIGIDYARNSVILRFQKHGRRQRLKYDPELEAKRRSLQDNFMHFGKRQAEQMPPEESYGADEEEGMVKRSDMDRYSRDPKQDFMRFGRVPKQDFMRFGRDPKQDFMRFGRDPSDFMRFGRAPSDFMRFGRTPSDFMRFGRTPSDFMRFGRSDNFMRLGRSPQQELRSPKQDFMRFGRPDNFMRFGRSAPPEFERYGKMDSNFMRFGKSSNPAAPPASGAPDSNQTKSQPASNPPGERSPVDKAMTLLFRKQEQQRQSKSGEQTEHTAEGGSVEQEQFYGQ

>Dsec FMRF dsec_GLEANR_3966

MGIALMFLLALYQMQSAIHSEIIDTPNYAGNSLQDADSEVSSPQDNDLVDALLGNDQTERAELEFRHPISVIGIDYSKNAVVLHFQKHGRKPRYKYDPELEAKRRSVQDNFMHFGKRQAEQLPPEGTYGGSDELEGMAKRVAMDRYGRDPKQDFMRFGRDPKQDFMRFGRDPKQDFMRFGRDPKQDFMRFGRDPKQDFMRFGRTPAEDFMRFGRTPAEDFMRFGRSDNFMRFGRSPHEELRSPKQDFMRFGRPDNFMRFGRSAPQDFVRSGKMDSNFIRFGKSVKPAAPESKPAKPNQGNPGERSPVDKAMTELFKKQELQQVKNGAQETTTEDGSVEQDQFFGQ

>Dsim FMRF dsim_GLEANR_10677

MGIALMFLLALYQMQSAIHSEIIDTPNFAGNSLQDADSEVSSPQDNDLVDALLGNDQTERAELEFRHPISVIGIDYSKNAVVLHFQKHGRKPRYKYDPELEAKRRSVQDNFMHFGKRQAEQLPPEGTYGGSDELEGMAKRAAMDRYGRDPKQDFMRFGRDPKQDFMRFGRDPKQDFMRFGRDPKQDFMRFGRDPKQDFMRFGRTPAEDFMRFGRTPAEDFMRFGRSDNFMRFGRSPHEELRSPKQDFMRFGRPDNFMRFGRSAPQDFVRSGKMDSNFIRFGKSVKPAAPESKPTKPNQGNPGERSPVDKAMTELFKKQELQDQQVKNGAQATTTEDGSVEQDQFFGQ

>Dvir FMRF dvir_GLEANR_7766

MGIALMFLLALYQMQSAIHSEIIETPSSYNDNSLLEAAAEEPNSRATASESDLLDGLMSTDNPNPEQQTELEFRYPISAIGIDYAKNSVVLRFQKHARKQNFKYDPDYEMKRKSLQDNFMHFGKRQAEQLPQATGPGYYEAVKRAAMDRYGRDPKQDFMRFGRAPPSDFMRFGRAPSDFMRFGRDPSQDFMRFGRSDNFMRFGRNLNFHEELRSPKQDFMRFGRPDNFMRFGRSAPTEFERNGKMDSNFMRFGKRSGVMAKLTKSQLQQNKLTTADGKQQPAEEGNPTDKAISMLFNKHQQQQQQQGQQLQQQQGQRLQQEERQQMKSSAEQNNLEEASVEQFYEP

>Dwil FMRF dwil_GLEANR_15987

MGIALMFLLALYQMQSAIHSEVIETPYNANALISEPEEDTQDTQVPVLADNGLIDSLLNEKPEQTELEFRYPISAIGIDYGKNSVVLRFQKNARKQRYKFDPEMEAKRRSLQDNFMHFGKRQPEQLPLEPQGNAYYEPETKRSPMDRYGRDPKQDFMRFGRDPKQDFMRFGRDPKQDFMRFGRDPKDFMRFGRTPSDFMRFGRTPSDFMRFGRADNFMRFGRSPHEELRSPKQDFMRFGRPDNFMRFGRSAPPDFEQFGKMDSNFIRFGKSVNAAAPAVTPEANQTKSQSQTKDKTGNPMNKAMSMLFTKEQPSKSSEQTATGSGDNSDDTSVEQFYGP

>Dyak FMRF dyak_GLEANR_3089

MGFALMFLLALYQMQSAIHSEIIDTPNYAGNSLQDTDSEVSSPLDNDLVDALLGDDQTERAELEFRHPISVIGIDYSKNAVVLHFQKHGRKPRYKYDPELEAKRRSVQDNFMHFGKRQAEQLPPEGSYGGSDELEGMSKRAGMDRYGRDPKQDFMRFGRDPKQDFMRFGRDPKQDFMRFGRDPKQDFMRFGRDPKQDFMRFGRTPAEDFMRFGRTPAEDFMRFGRSDNFMRFGRSPHEELRSPKQDFMRFGRPDNFMRFGRSAPQDFVRSGKMDSNFIRFGKSVKPAAPESKQIKPNQGNPGERSPVDKAMTELFKKQELQDQQVKATTTEDGSVERDQFFGQ

**hugin (HUGIN-pyrokinin)**

>Dana hug >dana_GLEANR_7892

MCGPSYCTLLLIAASCYVLVCSQAKSLQGTSKLDLANHISATSARGSSGPGTGTGPAAASASAAAARTSMNEARHKRAMGEYKELTDIIDELEENSLAQKASATLPLNAVPPQGQEFDLESMPPLTYYLLLQKLRQLQSSGEPAYRVRTPRLGRSIDFQQLLDGGDGGGGCRGLGVGVALAGSEEATGGQFMSRMIKKSVPFKPRLGKRAQVCGGGGD

>Dere hug dere_GLEANR_384

MCGPSYCTLLLIAASCYILVCSHAKSLQGTSKLDLGNHISAGSARGSSSPLASAALSEARQKRAMGDYKELTDIIDELEENSLAQKASATLQVAAVPPQGQEFDLDSMPPLTYYLLLQKLRQLQSNGEPAYRVRTPRLGRSIDSWRLLDAEGATGMAGGEEAIGGQFMQRMVKKSVPFKPRLGKRAQVCGGD

>Dgri hug dgri_GLEANR_3052

MFRLSNCTLLLIAASCYLVYSSQAKALQDANKLDLANRISGISAVSARTAAAAEARHKRAMGAEYKELNDIIDELEENSLVQKANAAVSPTQSQEFDLDNMPPLTYYLLLQKLRQLQSSGEPAYRVRTPRLGRSIDFQQFLGSGRGGSEEATGGQFVSRMMKKSVPFKPRLGKRAQVCGGGD

>Dmel hug CG6371

MCGPSYCTLLLIAASCYILVCSHAKSLQGTSKLDLGNHISAGSARGSLSPASPALSEARQKRAMGDYKELTDIIDELEENSLAQKASATMQVAAMPPQGQEFDLDTMPPLTYYLLLQKLRQLQSNGEPAYRVRTPRLGRSIDSWRLLDAEGATGMAGGEEAIGGQFMQRMVKKSVPFKPRLGKRAQVCGGD

>Dmoj hug dmoj_GLEANR_9611

MYRLSNCTLLLIAVSCYLVYASQAKALQGTNKLDLPNRISGINVGSAGAAAAAEARHKRAMGEYKELNDIIDDLDENGLVQKGGAAATASQAQAQEFDLDSMPPLAYYMLLERLRQWQRNNEQAYTRRTPRLGRSIDFQQLLDSRGGNEEAAGGQFVSRMMKKSVPFKPRLGKRAQVCGSGGD

>Dper hug dper_GLEANR_14374

MCRPSYGSLLLIAASCYVLVSSQAKALQGANKLDLANHISAGPARGSSAGGGADAVRAAMTEARHKRAMGEYKELTDIIDELEENSLSQKAAAVAPLPPQAQEFDLDSMPPLTYYLLLQKLRQSLTSSSSWTPGGASGEAAGGQFMSRMMKKSVPFKPRLGKRAQVCGSAGSGGD

>Dpse hug dpse_GLEANR_4915

MCRPSYGSLLLIAASCYVLVSSQAKALQGANKLDLANHISAGPARGSSAGGGAEAVRAAMTEARHKRAMGEYKELTDIIDELEENSLSQKAAAVAPLPPQAQEFDLDSMPPLTYYLLLQKLRQLQSNGEPAFRVRTPRLGRSIDFQQLLDAGGASGEAAGGQFMSRMMKKSVPFKPRLGKRAQVCGSAGSGGD

>Dsec hug dsec_GLEANR_7056

MCGPSYCTLLLIAASCYILVCSHAKSLQGTSKLDLGNHISAGSARGSSSATSPALSEARQKRAMGDYKELTDIIDELEENSLAQKTSATMQVAAMAPQGQEFDLDSMPPLTYYLLLQKLRQLQSNGEPAYRVRTPRLGRSIDSWRLLDAEGATGMSGGEEAIGGQFMQRMVKKSVPFKPRLGKRAQVCGGD

>Dsim hug dsim_GLEANR_2652

MCGPSYCTLLLIAASCYILVCSHAKSLQGTSKLDLGNHISAGSVRGSSSAASPALSEARQKRAMGDYKELTDIIDELEENSLAQKASATMQVAAMPPQGQEFDLDSMPPLTYYLLLQKLRQLQSNGEPAYRVRTPRLGRSIDSWRLLDAEGATGMAGGEEAIGGQFMQRMVKKSVPFKPRLGKRAQVCGGD

>Dvir hug dvir_GLEANR_10475

MYRLSNCTLLLIAASCYLVYSSHAKALQGTNKLDLPNRISDISVGSASAASAAEARHKRAMGEYKELNDIIDELEENNLGQKPSGAAPPAQTQEFDLDNMPPLAYYLLLQKLRQLQSSSEPTYRVRTPRLGRSIDFQQLLESRAGSEESTGGRFVSRMMKKSVPFKPRLGKRAQVCGGGD

>Dwil hug dwil_GLEANR_14044

MCRPRLCTLVLIAASCYALAISGNAKAMQGTNKLDLPNRISGEGRGSSFGTPATGARAGAGAGALVPQSKETRQKRAMGEYKELVDIIDELEENSLAQKTQSVAVPPPQTQEFDLDTMPPLTYYLLLQKLRQLQSNGEPAYRVRTPRLGRSIDFQQLLGEGGGAGADEASGGQFMSRMIKKSVPFKPRLGKRALMCGED

>Dyak hug dyak_GLEANR_9767

MCGPSYCTLLLIAASCYILVCSHAKSLQGTSKLDLGNHISVGSARGSSSPASAALSEARQKRAMGDYKELTDIIDELEENSLAQKASATMQVAAVPPQGQEFDLDSMPPLTYYLLLQKLRQLQSNGEPAYRVRTPRLGRSIDSWRLLDAEGATGMAGGEEAIGGQFMQRMVKKSVPFKPRLGKRAQVCGGD

**IFamide**

>Dana IFa dana_GLEANR_11310

MALRFTVTLLLVTILVAAILLGSSEAAYRKPPFNGSIFGKRNSLDYENPPSDYDSAKMSAVCEVAMEACPMWFPQNDSK

>Dere IFa dere_GLEANR_4758

MALRFALTLLLVTILVTAILLGSSEAAYRKPPFNGSIFGKRNSLGKPEIRIPVWIPNSKHPHSLPDYDSAKMSAVCEVAMEACPMWFPQNDSK

>Dgri IFa dgri_GLEANR_6010

MAALRCTLTLLLVTIIVAAILLHGSEAAYRKPPFNGSIFGKRNALDYDNAKAMGAACEVAVEACQSWFPQNDSK

>Dmel IFa CG33527 AF376801

MALRFTLTLLLVTILVAAILLGSSEAAYRKPPFNGSIFGKRNSLGKSKIRIPLKPPPISPSRLRQRQNERRLRGGHGGVSHVVSPERQQIGPRPATPPPRTDLEPTTNTPATGGQMLCLLVRLNVEMPDVKKVMYKIYNVSRAYRYIELMPYIYIKYSINLQH

>Dmel IFa Genscan predicted

MALRFTLTLLLVTILVAAILLGSSEAAYRKPPFNGSIFGKRNSLDYDSAKMSAVCEVAMEACPMWFPQNDSK

>Dmoj IFa dmoj_GLEANR_5249

MAALRCTLTLLLVTIIVAAILLHSSEAAYRKPPFNGSIFGKRNALGESAYHTYHISITISMRYSLDYDNAKAMSAVCEVAMEACQSWFPQNDSK

>Dper IFa dper_GLEANR_10168

MALRFTLTLLLVTILATAILLSSSEAAYRKPPFNGSIFGKRNGLDYDNAKMSAVCEVAMEACPMWFPQNDSK

>Dpse IFa dpse_GLEANR_16298

MALRFTLTLLLVTILATAILLSSSEAAYRKPPFNGSIFGKRNGLDYDNAKMSAVCEVAMEACPMWFPQNDSK

>Dsec IFa dsec_GLEANR_12173

MALRFTLTLLLVTILVAAILLGSSEAAYRKPPFNGSIFGKRNSLDYDSAKMSAVCEVAMEACPMWFPQNDSK

>Dsim IFa dsim_GLEANR_8942

MALRFTLTLLLVTILVAAILLGSSEAAYRKPPFNGSIFGKRNSLDYDSAKMSAVCEVAMEACPMWFPQNDSK

>Dvir IFa dvir_GLEANR_7425

MAALRCTLTLLLVTIIVAAILLHSSEAAYRKPPFNGSIFGKRNALDYDNAKAMGAVCEVAVEACQGWFPQNDSK

>Dwil IFa dwil_GLEANR_18781

MALRFTLTLLLVTILVAAILLGSSEAAYRKPPFNGSIFGKRNSLDYDSAKMSAVCEVAMEACPMWFPQNDSK

>Dyak IFa dyak_GLEANR_11770

MALRFTLTLLLVTILVTAILLGSSEAAYRKPPFNGSIFGKRNSLGKQKIRIPVEIPYSKHPYSPADYDSAKMSAVCEVAMEACPMWFPQNDSK

**Drosokinin**

>Dana kinin dana_GLEANR_9872

MQTWPLLILFLALGRHIYGASVAAPAPGLDTELGTCEIQLSKYRRFILQAILSFEDVCDAYNARPGGQEDTEGWIFRHYAPPPTSQRGEIWAFFRLLMAQFSDAEFTSIIRDAVIERCRIKSQLQRDEKRNSVVLGKKQRFHSWGGKRSSSPSESDY

>Dere kinin dere_GLEANR_13961

MANIVLCILLLVFGRQIYGASVASAPLSGQDPELATCELQLSKYRRFILQAILSFEDVCDAYNSRPGGQEADSEAWLFRHYAPAPTSQRSEIWAFFRLLMAQFGDAEFSPIIRDAVIERCRIKSQLQRDEKRNSVVLGKKQRFHSWGGKRSPEPPILPDY

>Dgri kinin dgri_GLEANR_16469

MLPIWRLLLLLSICRQLYALPTPHTSSSGGSTDVQLELNTCELQLAKYRKFILQAILSFEDVCDAYTARPVTSDDAQLADMWPFHQYAPPPTSQRGEIWAFFKLLMGQFNDMEFATIIRDAVIERCRIRVQRDDKRNSVVLGKKQRFHSWGGKRTPGQLSTNGGPLLTFPEAVSAAVNDAGLEHNYYN

>Dmel kinin CG13480

MAKIVLCMVLLAFGRQVYGASLVPAPISEQDPELATCELQLSKYRRFILQAILSFEDVCDAYSSRPGGQDSDSEGWPFRHYAPPPTSQRGEIWAFFRLLMAQFGDKEFSPIIRDAVIERCRIKSQLQRDEKRNSVVLGKKQRFHSWGGKRSPEPPILPDY

>Dmoj kinin dmoj_GLEANR_13714

MLSTWRLLLLLLGTCSPLYALPTHTATDLELSTCELQLGKYRRFILQAILSFEDVCDAYNSRRLAPDDGPGTSDTWLFDQYAPPPTSQRGEIWAFFKLLMAQFNDLEFASIIRDAVIERCRLKVQRDDKRNSVVLGKKQRFHSWGGKRSPDQASTLSLADAVAGAGMEHNLY

>Dper kinin dper_GLEANR_2876

MQTWRLLFLLLALSRQLYALPGHPASADVELNTCELQLSKYRRFILQAILSFEDVCDAYNAQSVSAEDRSPSEGGWLFHHYAPPPTSQRGEIWAFFKLLMAQFNDVEFASIIRDAVIERCRIKSQLQRDEKRNSVVLGKKQRFHSWGGKRSPEPLFAVPDPAVGAGPGTGVDRSYY

>Dpse kinin dpse_GLEANR_12344

MQTWRLLFLLLALSRQLYALPGHPASADVELNTCELQLSKYRRFILQAILSFEDVCDAYNARTVSAEDSSPSEGGWLFHHYAPPPTSQRGEIWAFFKLLMAQFNDVEFASIIRDAVIERCRIKSQLQRDEKRNSVVLGKKQRFHSWGGKRSPEPLFAVPDPGVGAGPGTGVDRSYY

>Dsec kinin dsec_GLEANR_7540

MAKIVLCMVLLAFGRQVYGASLAPAPISGQDPELATCELQLSKYRKFILQAILSFEDVCDAYSSRPGGQDADSEVWLFRHYAPPPTSQRGEIWAFFRLLMAQFGDADFSPIIRDAVIERCRIKSQLQRDEKRNSVVLGKKQRFHSWGGKRSLEPPILPDY

>Dsim kinin dsim_GLEANR_12679

MAKIVLCMVLLAFGRQVYGASLAPAPISGQDPELATCELQLSKYRKFILQAILSFEDVCDAYSSRPGGQDADSEVWLFRHYAPPPTSQRGEIWAFFRLLMAQFGDADFSPIIRDAVIERCRIKSQLQRDEKRNSVVLGKKQRFHSWGGKRSPEPPILPDY

>Dvir kinin dvir_GLEANR_11706

MLPTWRLLLLLAICRQLYALPAHTSVDLELNTCELQLAKYRKFILQAILSFEDVCDAYNARTVPPEDAPLSETWLFHQYAPPPTSQRGEIWAFFKLLMAQFNDVEFATIIRDAVIERCRFKLQRDDKRNSVVLGKKQRFHSWGGKRSPDQAVGGPLISLPEAVSGVAGTGMEHNYY

>Dwil kinin dwil_GLEANR_10795

MFSIWRLLLIVGLSRHLYATPITHLSSDQEQLGTCELQLTKYRKFILQAILSFEDVCDAYNSRPVNTEDGPLADGWIFRHYAPPPTSQRGEIWAFFKLLMAQFNDVEFATIIRDAVIERCRIKSQLQRDEKRNSVVLGKKQRFHSWGGKRSPEPLLFLQTDGAGQGVARLEHSYF

>Dyak kinin dyak_GLEANR_3867

MANIVLCVVLLAFGRQIYGASLASAPISGQDPELATCEVQLSKYRGFILQAVLSFEDVCDTYNSRPGGQDADSGAWLFRHYAPPPTSQRSEIWAFFRLLMAQFGDAEFSPIIRDAVVERCRIKSQLQRDEKRNSVVLGKKQRFHSWGGKRSPEPPILPDY

**Myoinhibiting peptides**

>Dana MIP dana_GLEANR_9049

MAYTKTRRNYGFLLGLLILGGACGTLTSGGSVASPVSAAEPGGPGGLTEQVVDQLSDGDLYGYSKRAWQSLQSGWGKRSDSGDPDIYMTGHFVPLVITDGTNTIDWETFERLASGQAAQQQQPQSQMQSGEDLDEIGADAEVEKRAWKSMNVAWGKRRQAQGWNKFRGAWGKREPTWNNLKGMWGKRDQWQKLHGGWGKRSAGSPLN

>Dere MIP dere_GLEANR_15860

MAHTKTRKTYGLLMVLLILGSACANLVASGSAASPASNEPGGGGLSEQVVLDQLSESDLYGNNKRAWQSLQSSWGKRSSSGDASDPDIYMTGHFVPLVITDGTNTIDWDTFERLASGPSAQQQQQQQEQQPLQQQSQSGEDFDDLAGESDVEKRAWKSMNVAWGKRRQAQGWNKFRGAWGKREPTWNNLKGMWGKRDQWQKLHGGWGKRSQLPSN

>Dgri MIP dgri_GLEANR_14752

MAQSLTSGGGTSSIGLVLLFLLCSTGSSQGAALTNNNNDMAATGLSDQVVEQLNDNDLFGNNNNKRAWQSLQSSWGKRSNDPAALEVDDSIYMTGHFVPLVITDGTNTIDWNTFERMASSSNLEQHQQPSQLQSQSNEDSDDNADNTLEKRAWKNMNVAWGKRRQAQGWNKFRGAWGKREPTWNNLKGMWGKRDQWQKLHAGWGKRSLAN

>Dmel MIP CG6456

MAHTKTRRTYGFLMVLLILGSACGNLVASGSAGSPPSNEPGGGGLSEQVVLDQLSESDLYGNNKRAWQSLQSSWGKRSSSGDVSDPDIYMTGHFVPLVITDGTNTIDWDTFERLASGQSAQQQQQQPLQQQSQSGEDFDDLAGEPDVEKRAWKSMNVAWGKRRQAQGWNKFRGAWGKREPTWNNLKGMWGKRDQWQKLHGGWGKRSQLPSN

>Dmoj MIP dmoj_GLEANR_12862

MAQSGISSSCATQSFGLLLLLLFLHSSRAAAMASGQSGNNNELAATGLTDQVVEQLGDNDLYNNKRAWQSLQSSWGKRSSSDPAALEMDDSIYMTGHFVPLVITDGTNTVDWNTFERWASNNQEQQLQQQQQQQQPSNSLEDADEFNSENGMEKRAWKNMNVAWGKRRQAQGWNKFRGAWGKREPTWNNLKGMWGKRDQWQKLHAGWGKRSLNN

>Dper MIP dper_GLEANR_16716

MAHTKTKTQNRILLVVVLLLCSACSSLATGSIASTASNEPSGLSEQVVDQMSDSDLYSNNKRAWQSLQSSWGKRSRDGPDGSEASFGGDDNIYMTGHFVPLVITDGTNTIDWDTFERLASNQDMQPQTLQQQQSGEDMVEDPEAAGNMEKRAWKSMNVAWGKRRQAQGWNKFRGAWGKREPTWNNLKGMWGKRDQWQKLHGGWGKRSMALN

>Dpse MIP dpse_GLEANR_13242

MVHTKTKTQNRILLVVVLLLCSACSSLATGSIASTASNEPSGLSEQVVDQMSDSELYSNNKRAWQSLQSSWGKRSRDGPDGSEASFGGDDNIYMTGHFVPLVITDGTNTIDWDTFERLASNQDMQPQTLQQQQSGEDMVEDPEAAGNMEKRAWKSMNVAWGKRRQAQGWNKFRGAWGKREPTWNNLKGMWGKRDQWQKLHGGWGKRSMALN

>Dsec MIP dsec_GLEANR_7349

MAHNKTRRTYGFLMVLLILGSACGNLVASGSAGSPPSNEPGGGGLSEQVVLDQLSESDLYGNNKRAWQSLQSSWGKRSSSGDASDPDIYMTGHFVPLVITDGTNTIDWDTFERLASGNGQSTQQQQQQPLQQQAQSGEDFDDLAGESDVEKRAWKSMNVAWGKRRQAQGWNKFRGAWGKREPTWNNLKGMWGKRDQWQKLHGGWGKRSQLPSN

>Dsim MIP dsim_GLEANR_12481

MAHTKTRRTYGFLMVLLILGSACGNLVASGSAGSPPSNEPGGGGLSEQMVLDQLSESDLYGNNKRAWQSLQSSWGKRSSSGDASDPDIYMTGHFVPLVITDGTNTIDWDTFERLASGNGQSAQQQQQQPLQQQSQSGEDFDDLAGESDVEKRAWKSMNVAXGKRRQAQGWNKFRGAWGKREPTWNNLKGMWGKRDQWQKLHGGWGKRSQLPSN

>Dvir MIP dvir_GLEANR_12958

MKSCVCGGTHGMALLLLLLLFWCRTSCSQAAAGGGNNNELAATGLSDQVLEQLDDNELYGNNKRAWQSLQSAWGKRSNEPRPLEVDDSIYMTGHFVPLVITDGTNTIDWNTFERLASGNQEQQQLQRQQQPAQSNEESEDVSAENTIDKRAWKNMNVAWGKRRQAQGWNKFRGAWGKREPTWNNLKGMWGKRDQWQKLHAGWGKRSLAN

>Dwil MIP dwil_GLEANR_3116

MAWKYSGGLMVLLLCTTASSSLNLASTTTTSNEPTGLSEQVVDQLSENDLYQANKRAWQSLQSSWGKRSNAGDVVGGFGAGGDDIYMTGHFVPLVVTDGTNTIDWKTFERMANQGQQQQGDQVAEQPQSDEEPDEIGDVEAASNNLLDKRAWKNMNVAWGKRRQAQGWNKFRGAWGKREPTWNNLKGMWGKRDQWQKLHGGWGKRSPMN

>Dyak MIP dyak_GLEANR_5860

MAHNKTRRSYGFLMVLLILGSACGSLVASGSAANSASNEPGGGGLSEQVVLDQLSESDLYGNNKRAWQSLQSSWGKRSSSGDASDPDIYMTGHFVPLVITDGTNTIDWDTFERLASGQPAQQQQQPLQQQSQSGEDFDDVAGDSDVEKRAWKSMNVAWGKRRQAQGWNKFRGAWGKREPTWNNLKGMWGKRDQWQKLHGGWGKRSQLPSN

**Myosuppressin**

>Dana MS dana_GLEANR_3988

MSFAQFFFACCLAVILLAVANTQAATQAPPLCQVGIVEEMPQHIRKVCQALENSDQLSSALKSYINNEASALVANSDDLLKNYNKRTDVDHVFLRFGKRR

>Dere MS dere_GLEANR_11394

MSFAQFFVACCLAIVLLAVSNTRAAAQGPPLCQSGIVEEMPPHIRKVCQALENSDQLTSALKSYINNEASALVANSDDLLKNYNKRTDVDHVFLRFGKRR

>Dgri MS dgri_GLEANR_3156

MSHVQFTAICCFAIVLLAVGVHSAQAATQAPLCQPGIVEEMPQHIKKVCMALENSDQLTSALKSYINNEASALVANSDDLMKNYNKRTDVDHVFLRFGKRR

>Dmel MS CG6440

MSFAQFFVACCLAIVLLAVSNTRAAVQGPPLCQSGIVEEMPPHIRKVCQALENSDQLTSALKSYINNEASALVANSDDLLKNYNKRTDVDHVFLRFGKRR

>Dmoj MS dmoj_GLEANR_10029

MSHVQLLAICFLAIVLLVVCAHSAATPAPLCQPGIVDEMPQHIKKVCLALENSDQLNTVLKSYINSEASALVANSEDLLKNYNKRTDVDHVFLRFGKRR

>Dper MS dper_GLEANR_3930

MSFAQFFVACCLAIVLAAVANTQAATQGPPLCQAGIVQEMPQHIQKVCMALENSDQLTSALKSYINNEASALVANSDDLLKNYNKRTDVDHVFLRFGKRR

>Dpse MS dpse_GLEANR_3565

MSFAQFFVACCLAIVLAAVANTQAATQGPPLCQAGIVQEMPQHIQKVCMALENSDQLTSALKSYINNEASALVANSDDLLKNYNKRTDVDHVFLRFGKRR

>Dsec MS dsec_GLEANR_9458

MSFAQFFVACCLAIVLLAVSNTRAAVQGPPLCQSGIVEEMPPHIRKVCQALENSDQLTSALKSYINNEASGELIRTRPLPLDYLTSLFAPALVANSDDLLKNYNKRTDVDHVFLRFGKRR

>Dsim MS dsim_GLEANR_4854

MSFAQFFVACCLAIVLLAVSNTRAAVQGPPLCQSGIVEEMPPHIRKVCQALENSDQLTSALKSYINNEASGELIRTRPLSLDYLTSIFAPALVANSDDLLKNYNKRTDVDHVFLRFGKRR

>Dvir MS dvir_GLEANR_9135

MSHAQSFAICCLAIVLLAVCVQSAEAASQAPLCQPGIFEEMPEHIKKVCMALENSDQLNSALKSYINNEASALVANSDDLLKNYNKRTDVDHVFLRFGKRR

>Dwil MS dwil_GLEANR_5550

MSSFVQFFVVSCFACIVLLLVPSSCQAATQATPLCQAGIVEEMPVHIKKVCMALENSDQLTSALKSYINNEASALVANSDDLLKNYNKRTDVDHVFLRFGKRR

>Dyak MS dyak_GLEANR_7258

MSFAQFFVACCLAIVLLAVSNTRAAVQGPPLCQSGIVEEMPPHIRKVCQALENSDQLTSALKSYINNEASDYLTFMSTAALVANSDDLLKNYNKRTDVDHVFLRFGKRR

**Neuropeptide F**

>Dana NPF dana_GLEANR_18326

MRCILIVCVAIALLAAGCSVEASNSRPPRKNDVNTMADAYKFLQDLDTYYGDRARVRFGKRGNLMELLRNREIENNFNLGKGVNSGGEL

>Dere NPF dere_GLEANR_650

MRCILVACVALALLAAGCRVEASNSRPPRKNDVNTMADAYKFLQDLDSYYGDRARVRFGKRGSLMEILRNHEMDNLNLGKGANNGGEL

>Dgri NPF dgri_GLEANR_452

MRCTLVVCVALALFAAGCNVEASKARPPRNNEISNMADALKYLQDLDVYYGDRARVRFGKRASTIQALHNRMLESGLNSNSNDNFNENNGGVL

>Dmel NPF CG10342

MRCILVACVALALLAAGCRVEASNSRPPRKNDVNTMADAYKFLQDLDTYYGDRARVRFGKRGSLMDILRNHEMDNINLGKNANNGGEFARGFNEEEIF

>Dmoj NPF dmoj_GLEANR_7585

MRCTLVICVAFALFAAGCIVEASKTRPPRNNEINTMADALKYLQDLDVYYGDRARVRFGKRGSLMQAIRNRMLLNGVNTEPFNDNNNGGEL

>Dper NPF dper_GLEANR_9805

MRCILIVCVALTLIAAGCNVEASNSRPPRKNDVNTMADAYKFLQDLDTYYGDRARVRFGKRGGPLMEMLRNRELENNMAKSINSGGEL

>Dpse NPF dpse_GLEANR_4391

MRCILIVCVALTLIAAGCNVEASNSRPPRKNDVNTMADAYKFLQDLDTYYGDRARVRFGKRGGPLMEMLRNRELENNMAKSINSGGEL

>Dsec NPF dsec_GLEANR_15982

MRCILVACVALALLAAGCRVEASNSRPPRKNDVNTMADAYKFLQDLDTYYGDRARVRFGKRGSLMEILRNHEMDNINLGKNVNNGGEL

>Dsim NPF dsim_GLEANR_2914

MRCILVACVALALLAAGCRVEASNSRPPRKNDVNTMADAYKFLQDLDTYYGDRARVRFGKRGSLMEILRNHEMDNINLGKNVNNGGEL

>Dvir NPF dvir_GLEANR_9674

MRCTLVVCVALALFAAGCNVEASKARPPRNNEISTMADALKYLQDLDVYYGDRARVRFGKRGSVMQAIRNRVLENGINSYNNENINDNGGEL

>Dwil NPF dwil_GLEANR_5544

MRCILIVCVAFALLATSCSVEASNSRPPRNNEINTMADAYKFLQDLDTYYGDRARVRFGKRNPMAQLFRNRMLDASNVNGANGELVSSIQLKVVDICKY

>Dyak NPF dyak_GLEANR_8263

MRCILVVCVALALLAAGCRVEASNSRPPRKNDVNTMADAYKFLQDLDSYYGDRARVRFGKRGSLMEILRNHEMDNLNLGKSANNGGELVTIE

**Neuropeptide-like precursor 1**

>Dana NPLP1 dana_GLEANR_13375

MQAVHKSAHHSKRLLLLLSMLLNAALQPRSFAASATEDVANVSPCEMESIINQLMNPSPEYQLHASALRNQLKNLLRERQLAVGEEQPLGDYSDYVEEDKRSVAALAAQGLLNASPKRSLATLAKNGQLPTAEPGEDYADADSGEPSEQKRYIGSLARAGGLMTYGKRNVGTLARDFQLPIPNGKRNLAMVARLQSAPATHREQPKRNVAAVARYNSQQHHNQRASAEKRNLGALKSSPVHGAQQKREDEEMLLPAAAPDYADPMQSYWWYPSYAGYADLDWNDYRRAEKRFLDTSKDPELFGIEHGNDAALQQEEEADDPGQAAPGTEPEEQWPSPQKRHIGAVYRSGFLPSYRYLRSPTPGGGYGGGAGGRFSRSGRDARQFVGYFPQHERLRQPTAAGCKQCFLPNHQPMITWSGAGIRGRLSRLAPGRSEHFRSLSSNALPSGGSPRSLLRSGAPSYPPFHAWGTPPRITALHRREFRRNMDNYEY

>Dere NPLP1 dere_GLEANR_7703

MQAVLQSAHSSRRLMLLLSMLLNAAIQPHSIIVSATDDVANVSPCEMESIINQLMNPSPEYQLHASALRNQLKNLLRERQLAVGEEQPLGEYSDYMEEDKRSVAALAAQGLLNAPKRSLATLAKNGQLPTAEPGEDYADADSGEPSEQKRYIGSLARAGGLMTYGKRNVGTLARDFQLPIPNGKRNIATMARLQSAPSTHRDPKRNVAAVARYNSQHGNIQRAGAEKRNLGALKSSPVHGVQQKREDEEMLLPAAAPDYADPMQSYWWYPSYAGYADLDWNDYRRAEKRFLGRVLPPTRATASTHRSRL

>Dgri NPLP1 dgri_GLEANR_6993

MQDVHNTAPRSKWLLLLFSMLLNAAVQPRAFNVNASEDIGNVSPCEMESIINQLMNPGPEYQLHASALRNQLKNLLRERQLAVGEEQPLGDYADYLDEDKRSVAALAAQGLLTPKRSLATLAKNGQLPTAEPGDDYADADSGEPSEQKRYIGSLARAGGLMSFGKRNVGTLARDFQLPNGKRNIATMARLQSAPSTHREPPKRNVAAVARYNSQQHQNHQRSNGEKRNLGALKSSPVHGMMQQKREDVEMALLQPAAAASAAAPDYADPMQSYWWYPSYAGYADLDWNDYRRAEKRYLDTSKDPELFGIEHGNEATNLANFPDLQMEPEQEQEQELEREHEREQPEQPPEKRHIGAVYRSGFLPSYRYLRSPTMNGAGGRFSRSGRARQFVEYYPQHERLRQPIAAVCKRCFLPNRPMMNWSSAGIRGSLSKYYTDPLSRSSTAATAARMRSSSSSILTNSLPSGHHNNISNSNRNRIYPPFHAWGTPPRITALQRRVYRRNGESMDNYEY

>Dmel NPLP1 CG3441-RA

MQAVLQSAHSSRRLMLLLSMLLNAAIQPRSIIVSATDDVANVSPCEMESLINQLMSPSPEYQLHASALRNQLKNLLRERQLAVGEEQPLGEYPDYLEEDKRSVAALAAQGLLNAPKRSLATLAKNGQLPTAEPGEDYGDADSGEPSEQKRYIGSLARAGGLMTYGKRNVGTLARDFQLPIPNGKRNIATMARLQSAPSTHRDPKRNVAAVARYNSQHGHIQRAGAEKRNLGALKSSPVHGVQQKREDEEMLLPAAAPDYADPMQSYWWYPSYAGYADLDWNDYRRAEKRFLGRVLPPTRATASTHRSRL

>Dmel NPLP1 spliceB

MQAVLQSAHSSRRLMLLLSMLLNAAIQPRSIIVSATDDVANVSPCEMESLINQLMSPSPEYQLHASALRNQLKNLLRERQLAVGEEQPLGEYPDYLEEDKRSVAALAAQGLLNAPKRSLATLAKNGQLPTAEPGEDYGDADSGEPSEQKRYIGSLARAGGLMTYGKRNVGTLARDFQLPIPNGKRNIATMARLQSAPSTHRDPKRNVAAVARYNSQHGHIQRAGAEKRNLGALKSSPVHGVQQKREDEEMLLPAAAPDYADPMQSYWWYPSYAGYADLDWNDYRRAEKRFLDTSKDPELFGIEHGNDATTAEPADEAYMESDAEAGSEQLPSPQKRHIGAVYRSGFLPSYRYLRSPGGSSGFGGAGGRFSRSGRDARQFV

>Dmoj NPLP1 dmoj_GLEANR_14312

MQAVHTNAQRSQWLLLLFAMLLNAAVQPRDFAVNAAEDIGNVSPCEMETIINQLLNPSPEYQLHASALRNQLKNLLREREEQQQQGDYGDYLDEDKRSVAALAAQGLLNAPKRSLATLAKNGQLPTAEPGDDYADAESGEPSEQKRYIGSLARAGGLMSFGKRNVGTLARDFQLPNGKRNVATMARLQSAPSTHREQPKRNVAAVARYNSQQHQNQRNSGEKRNLGALKSSPVHGVQQKREDEEMLLPAVAAAAPDYADPMQSYWWYPSYAGYADLDWNDYRRAEKRFLDTSKDPELFGIEHGNEATDADANVAALAEELEEDQQQQQQQQAALQAMPQKRHIGAVYRSGFLPSYRYLRSPTGNTGGGFGGAGGRFSRSGRARQLVEYYPQHERLRQPIAAVCKRCFLPNRPMMNWSGAGIRGSLSKFYVDPIVETARSSAANAARIRSISTNSLPSGPSKSRIYPFHSWGTPPRITALQRGVYRRNGESMDNYEY

>Dper NPLP1 dper_GLEANR_19435

MQAVHKTAHSSKWLILLLSMLLNAAFQPRSFAVSATDDVANVSPCEMESIISQLMNPSPEYQLHASALRNQLKNLLRERQLAVGEEQPLGEYSDYMEEDKRSVAALAAQGLLNAPKRSLATLAKNGQLPTAEPGEDYADADSAEPSEQKRYIGSLARAGGFMTYGKRNVGTLARDFQLPMPNGKRNLAMVARLQSAPSTHREQPKRNVAAVARYNSQQHHNQRAGAEKRNLGALKSSPVHGVQQKREDEEMMLPAAAPDYADPMQSYWWYPSYAGYADLDWNDYRRAEKRFLDTSKDPELFGIEHGNDAELNDAVAVAAADADADAEEPDTEPGNEQSTSPQKRHIGAVYRSGFLPSYRYLRSTTGGGGGRFSRSGRDARQFVAYYPQHERLRQPAAVCKRCFLPNQPTMNWSGAGIRGRFPQQFEGESPSPSAFSAYMRSTSSNSLPTGSTRSLKRPLSRSGATVYPPFHSWGTPPRITALHRREFRRNMDNYEY

>Dpse NPLP1 dpse_GLEANR_15771

MQAVHKTAHSSKWLILLLSMLLNAAFQPRSFAVSAADDVANVSPCEMESIISQLMNPSPEYQLHASALRNQLKNLLRERQLAVGEEQPLGEYSDYMEEDKRSVAALAAQGLLNAPKRSLATLAKNGQLPTAEPGEDYADADSAEPSEQKRYIGSLARTGGFMTYGKRNVGTLARDFQLPMPNGKRNLAMVARLQSAPSTHREQPKRNVAAVARYNSQQHHNQRAGAEKRNLGALKSSPVHGVQQKREDEEMMLPAAAPDYADPMQSYWWYPSYAGYADLDWNDYRRAEKRFLDTSKDPELFGIEHGNDAELNDAVAVAAADADADAEEPDTEPGNEQSTSPQKRHIGAVYRSGFLPSYRYLRSTTGGGGGRFSRSGRDARQFVAYYPQHERLRQPAAVCKRCFLPNQPTMNWSGAGIRGRFPQQFEGESPSPSAFSAYMRSISSNSLPTGSTRSLKRPLSRSGAPVYPPFHSWGTPPRITALHRREFRRNMDNYEY

>Dsec NPLP1 dsec_GLEANR_12268

MQAVLQSAHSSRPLMLLLSMLLNAAIQPQSVIVSATDDVANVSPCEMESLINQLMSPSPEYQLHASALRNQLKNLLRERQLAVGEEQPLGEYPDYLEEDKRSVAALAAQGLLNAPKRSLATLAKNGQLPTAEPGEDYADADSGEPSEQKRYIGSLARAGGLMTYGKRNVGTLARDFQLPIPNGKRNIATMARLQSAPSTHRDPKRNVAAVARYNSQHGQIQRAGAEKRNLGALKSSPVHGVQQKREDEEMLLPAAAPDYADPMQSYWWYPSYAGYADLDWNDYRRAEKRFLDTSKDPELFGIEHGNDATAAEPADEAEMESDAEAGSEQLPSPQKRHIGAVYRSGFLPSYRYLRSPGGSGGFGGAGGRFSRSGRDARQFAGYFPQHERLRQPTAAVCKQCFIPNQPMINWSGAGVRGRLSKYYVDPESSLARLPSLSSNSLPSGRPPRPLLRSGAPVYPPFHTWGTPPRITALHRREFRRNSENYEY

>Dsim NPLP1 dsim_GLEANR_11869

MQAVLQSAHSSRRLMLLLSMLLNAAIQPQSVIVSATDDVANVSPCEMESLINQLMSPSPEYQLHASALRNQLKNLLRERQLAVGEEQPLGEYPDYLEEDKRSVAALAAQGLLNAPKRSLATLAKNGQLPTAEPGEDYADADSGEPSEQKRYIGSLARAGGLMTYGKRNVGTLARDFQLPIPNGKRNIATMARLQSAPSTHRDPKRNVAAVARYNSQHGQIQRAGAEKRNLGALKSSPVHGVQQKREDEEMLLPAAAPDYADPMQSYWWYPSYAGYADLDWNDYRRAEKRFLDTSKDPELFGIEHGNDATAAEPADEAEMESDAEAGSEQLPSPQKRHIGAVYRSGFLPSYRYLRSPGGSGGFGGAGGRFSRSGRDARQFV

>Dvir NPLP1 dvir_GLEANR_6416

MQSVHKTAHRSKWFLLLLSMLLNAAVQPRAFAVKAAEDIGNMSPCEMETIINQLMNPSPEYQLHASALRNQLKNLLRERQLAVGEEQPLGDYADYLDEDKRSVAALAAQGLLNTPKRSLATLAKNGQLPTSEPGDDYADAESGEPSEQKRYIGSLARAGGLMSFGKRNVGTLARDFQLPNGKRNIATMARLQSAPGTHRDQPKRNVAAVARYNSQQHQNQRNSAEKRNLGALKSSPVHGMQQKREDEEMLLMPAVAAAAPDYADSMQNYWWYPSYAGYADLDWNDYRRAEKRFLDTSKDPELFGIEHGNEATDAGAAVLAEELEEEQQQQQATPQKRHIGAVYRSGFLPSYRYLRSPTGNTAGGYGGAGGRFSRSGRARQFVGYYPQHERLRQPIAAVCKRCFLPNRPMMNWSGAGIRGSLSKIYADPIEAARSSAATAARMRSISTNSLPSGPAKSNNRPIYPPFHSWGTPPRITALHRGVYRRNGESMDNYEY

>Dwil NPLP1 dwil_GLEANR_4683

MQAVHKIAQTNHGKSMMLILSMLLLNAAFEMRSFGVSAAEDDGTGNVSPCELESIINQLMNPSPEYQLHASALRNQLKKLLRERQQSVGEEQLLGDGYMEEDKRSVSSLAAQGLLNPPKRSLATLAKNGQLPTVEPGEDYVDADSGEPSSEQKRYIGSLARAGGLMTYGKRNVGTLARDFQLPTGKRNLAMLARLQSAPTSHREQPKRNVAAVARYNSQQHHNQRGASAEKRNLGALKSSPVHGAQQKREDEEILLPAAAPDYADPMQSYWWYPSYAGYADLDWNDYRRAEKRFLDTSKDPELFGIEHGYDATAISEEPLAAAAREEKEEEEEQLELQQPLPQKRHIGAVYRSGFLPSYRYLRSPTVTGSYGGAGGRFSRSGRDARQFVGYYYPQQERLRQPIAAVCKRCFLPNQPMINWNSAAGIRGRLPNKYNTNTNYPESEDSWRSINTNSLPTNRAQMRSGLTTSRTYPPFHSWGTPPRITALLHRRELRGNNMDNFE

>Dyak NPLP1 dyak_GLEANR_14581

MQAVLQSAHSSRRLMLLLSMLLNAAIQPHSIIVSATDDVANVSPCEMESLINQLMNPSPEYQLHASALRNQLKNLLRERQLAVGEEQPLGEYTDYLEEDKRSVAALAAQGLLNAPKRSLATLAKNGQLPTAEPGEDYADADSGEPSEQKRYIGSLARAGGLMTYGKRNVGTLARDFQLPIPNGKRNIATMARLQSAPSTHRDPKRNVAAVARYNSQQGHIQRAGAEKRNLGALKSSPVHGVQQKREDEEMLLPAAAPDYADPMQSYWWYPSYAGYADLDWNDYRRAEKRFLDTSKDPELFGIEHGNDATAAAPADEAEMESDADADGEELPSPQKRHIGAVYRSGFLPSYRYLRSPGGGAGFGGAGGRFSRSGRDARQFAGYFPQHERLRQPTAAVCKQCFIPNQPMINWSSAGVRGRLSKYYVDPEPSSARLPSLSTNSLPSGRPPRPLLRSGAPVYPPFHTWGTPPRITALHRREFRRNSENYEY

**Pigment-dispersing factor**

>Dana_PDF dana_GLEANR_20137

MARCTFTLVLLLLAVCFHCGMALALPDEERYVRKEYNRDLLDWFNNMGQFTPGQVATLCRYPLILENSLAGPMPIRKRNSELINSLLSLPKNMNDAGK

>Dere_PDF dere_GLEANR_12230

MACYTYLAALVLLVICCQWGSCAALAMPDEERYVRKEYNRDLLDWFNNVGVGVGQFTPGQVATLCRYPLIVENSLGPSMPIRKRNSELINSLLSLPKNMNDAGK

>Dgri_PDF dgri_GLEANR_14033

MAHYVVALSLLLVVASCSSVLSAPDEERYVSKDYNRDLYDWLTATRYAVAGQPYKYPYYLGNSVASNMRLPKRNSELINSLLSLPKNMNEAGK

>Dmel_PDF CG6496

MARYTYLVALVLLAICCQWGYCGAMAMPDEERYVRKEYNRDLLDWFNNVGVGQFSPGQVATLCRYPLILENSLGPSVPIRKRNSELINSLLSLPKNMNDAGK

>Dmoj_PDF dmoj_GLEANR_10577

MAHNSLTVALLALACCLCWSLVEASPDEERYVEKEYNRDLYDWINNVARYGRVQMPATLCKYPNYLANSLTNNMRMPKRNSELINSLLSLPKNMNEAGK

>Dper_PDF dper_GLEANR_6492

MAQYALTLAMLIAALCSRCELSRTMSLPDEERYVRKEYNRDLMDWFNTVGQLAPNQGVPLCRYPYIADNSLSIPTRKRNSEIINSLLSLPKNMNEAG

>Dpse_PDF dpse_GLEANR_3842

MAQYALTLAMLIAALCSRCELSRTMSLPDEERYVRKEYNRDLMDWFNTVGQLAPNQGVPLCRYPYIADNSLSIPTRKRNSEIINSLLSLPKNMNEAG

>Dsec_PDF dsec_GLEANR_10212

MARYTYLVALVLLAICCQWGSGGAMAMPDEERYVRKEYNRDLLDWFNNVGVGQFSPSQVATLCRYPLILENSLGPSVPIRKRNSELINSLLSLPKNMNDAGK

>Dsim_PDF dsim_GLEANR_1964

MARYTYLVALVLLAICCQWGSCGAMAMPDEERYVRKEYNRDLLDWFNNVGVGQFSPGQVATLCRYPLILENSLGPSVPIRKRNSELINSLLSLPKNMNDAGK

>Dvir_PDF dvir_GLEANR_8380

MTCYALTLALLALAGCICCTFARATPDEERYVEKEYNRDLYDWINNAVRYAPVQPPGPPCKYPYFLDNSLNPNMRMPKRNSELINSLLSLPKNMNDAGK

>Dwil_PDF dwil_GLEANR_11574

MARYGMIFSLLILAICCHCCCCAALAAPDEERYIRKDFNRDLLDWYNNANQYPSGQLASLCRYPYFLDNSLIGPVRMRKRNSELINSLLSLPKNMNDAGK

>Dyak_PDF dyak_GLEANR_10538

MARYTYLAALVLLAICCQWGSCAAMAMPDEERYVRKEYNRDLLDWFNNVGVGVGQFSPGQVATLCRYPLILENSLGATMPIRKRNSELINSLLSLPKNMNDAGK

**Proctolin**

>Dana proc dana_GLEANR_731

MRAARSIDQQASPGDSRLMRGGCGNGSGNGHRWLLVWMMVLLLVVPSHLVDGRYLPTRSHGDDLDKLRELMLQVSKITLEILELSNEDPQTQQQQQHPLRLHNEATGNSNGNSIGSSSNINNPRVSNSNSNAAWLQKLSAMGALDELGGDAPRYGPNYGRY

>Dere proc dere_GLEANR_8313

MGVLRRQGTGIGSGSGSGHRWLLVWMTVLLLVVPPHLVDGRYLPTRSHGDDLDKLRELMLQILELSNEDPQQQQQQQQQQQQHPQLRLHNEAIGGSSSSSSSNSNNPRVSNGNSNAAWLQKLSAMGALDELGGDGVRFGPNYGRY

>Dgri proc dgri_GLEANR_11433

MSVIGNRASSHKLQLSLPLAVVVVVMMLLVVVPLQPCESRYLPTRSHGDELDKLRELMLQILELSNEDPQQQQQQQQHQMRLHNEANNPLTAQRVGNSNSNSNAAWLQKLGAMGALDTEAGYGRY

>Dmel proc CG7105

MGVPRSHGTGIGCGSGHRWLLVWMTVLLLVVPPHLVDGRYLPTRSHGDDLDKLRELMLQILELSNEDPQQQQQQQQQQQHPQLRLHNEATGGSSSSSNINNPRVSNGNSNAAWLQKLSAMGALDELGGDGARFGPNYGRY

>Dmoj proc dmoj_GLEANR_1846

MPSPRLQSSPSLSLSLPMPLLLSLMLLLLLLVPPQPCESRYLPTRSHGDELDKLRELMLQILELSNEDPQQQQQQQPHQMRLHNEANNPLTAQRGSNSGSSANAAWLQKLGAMGALDTDAGYGRY

>Dper proc dper_GLEANR_21619

MGMGLNLRQGHRWLVWLLLLLLAIPPQMVDGRYLPTRSHGDDLDKLRELMLQILELSNEDPQQQQQQQQQQTMPQQHPLLRLHNEANSGSSSTAGGSSNGNNPRVSNGNSNAAWLQKLSAMGALDELGGDAPRMGPNYGRY

>Dpse proc dpse_GLEANR_2523

MGMGLNLRQGHRWLVWLLLLLLAIPPQMVDGRYLPTRSHGDDLDKLRELMLQILELSNEDPQQQQQQQQQQTMPQQHPLLRLHNEANSGSSSTAGGSSNGNNPRVSNGNSNAAWLQKLSAMGALDELGGDAPRMGPNYGRY

>Dsec proc dsec_GLEANR_1387

MGVPRRQGTEIGCGSGHRWLLVWMTVLLLVVPPHLVDGRYLPTRSHGDDLDKLRELMLQVSQSTLGESFDASVNSFARKLILELSNEDPQQQQQQQQQHPQLRLRNEATGGSSSSNINNPRVSNGNSNAAWLQKLSAMGALDELGGDGARFNPNYGRY

>Dsim proc dsim_GLEANR_6211

MGVPRRQGTGIGCGSGHRWLLVWMTVLLLVVPPHLVDGRYLPTRSHGDDLDKLRELMLQILELSNEDPQQQQQQQQQHPQLRLRNEATGGSSSSSNINNPRVSNGNSNAAWLQKLSAMGALDELGGDGARFNPNYGRY

>Dvir proc dvir_GLEANR_1002

MTADRNQAQVQAQAQAEAPALATAPTLARSHRLQLSLPLPLLLALMMLLLLLVPPQPCESRYLPTRSHGDELDKLRELMLQILELSNEDPQQQQQQSHQMRLHNEANNPLTAQRASGSSNAAWLQKLGAMGALDTEGGYGRY

>Dwil proc dwil_GLEANR_7917

MKAARNQVYGPKVLIWLLLLLLAMPPQMVDGRYLPTRSHGDDLDKLRELMLQILELSNEDPQQQQQQQQQQPLQQQQQHPLLRLHNEGNNPLLANSQRGSNSNAAWLQKLSAMGALDEQGGGYGRY

>Dyak proc dyak_GLEANR_2117

MGVPRRQGTGIGVGSGSGSGHRWLLVWMTVFLLVVPSHLVDGRYLPTRSHGDDLDKLRELMLQVSNCTLGEIGNNIAGNSILELSNEDPQQQQQPQQQQQQQHPQLRLHNEATVGSSNSNSNSNINNPRVSNGNSNAAWLQKLSAMGALDELGGDGVRFGPNYGRY

**Sulfakinin**

>Dana SK dana_GLEANR_22325

MGFRTWSRLAVLAIPLWAVAFYLLVVMPVPGHTASLGSGKEEQRQQDLETKIGTDSEQSNGYSRDTPFHSRFSNRRNQRSAGFVHRLPIFSRPIIPIELDLIMDTDEEIRPKTKRFDDYGHMRFGKRGGDDQFDDYGHMRFGR

>Dere SK dere_GLEANR_1187

MQVPAQTTSLQISKEDRRLQELESKMGAESEQPNANLVGPSISRFGDRRNQKTISFGRRVPLISRPMIPIELDLLMDNDDERTKAKRFDDYGHMRFGKRGGDDQFDDYGHMRFGR

>Dgri SK dgri_GLEANR_551

MCYSSAFLLLGFTVYFFLVVPTLSHAGSVEPAKEELQLEPKLELESGHVPGPSLVHFGNSRRNLRSIGFGHRFFPITRSKIPIELEMLVENDEIERPKRFDDYGHMRFGKRNGDDQFDDYGHMRFGR

>Dmel SK CG18090

MGPRSCTHFATLFMPLWALAFCFLVVLPIPAQTTSLQNAKDDRRLQELESKIGGEIDQPIANLVGPSFSLFGDRRNQKTMSFGRRVPLISRPIIPIELDLLMDNDDERTKAKRFDDYGHMRFGKRGGDDQFDDYGHMRFGR

>Dmoj SK dmoj_GLEANR_10420

MLPYKSPGRCSGALILVVLAVYLLLSLPSPSHAESLDSLKEEQQRRNMEPKLESDSDTLNVNGAGTARTHFGHNNRRNQRSIGYGPRFFPISHSKIPIELELLVNNEEAERPKRFDDYGHMRFGKRGNDEQFDDYGHMRFGR

>Dper SK dper_GLEANR_12463

MGHRGMGCAHFATLAMPLWALTFYLLVVLPVPSQTASVEVGKEERRLQDLDPKMGSEAGNTDGLSLARFGSRRHQRSTGFGHRVPIISRPVIPIELDLLMDNEDDRTMSKRFDDYGHMRFGKRGGDDQFDDYGHMRFGR

>Dpse SK dpse_GLEANR_3189

MAMPLWALTFYLLVVLPVPSQTASVEVGKEERRLQDLDPKMGSEAGNTDGLSLARFGSRRHQRSTGFGHRVPIISRPVIPIELDLLMDNEDDRTMSKRFDDYGHMRFGKRGGDDQFDDYGHMRFGR

>Dsec SK dsec_GLEANR_10746

MGLRSCTHLATLFMTLWALAFCFLVVVPIPAQTTSLQNAKDDRRLQELESKIGAESDQPNANLVGPSFSRFGDRRNQKTISFGRRVPLISRPMIPIELDLLMDNDDERTKAKRFDDYGHMRFGKRGGDDQFDDYGHMRFGR

>Dsim SK dsim_GLEANR_3491

MGLRSCTHLATLFMTLWAVAFCFLVVVPIPAQTTSLQNAKDDRRLQELESKIGAESDQTNANLVGPSFSRFGDRRNQKAISFGRRVPLISRPMIPIELDLLMDNDDERTKAKRFDDYGHMRFGKRGGDDQFDDYGHMRFGR

>Dvir SK dvir_GLEANR_8538

MLPNWSPKWYSSALTLAVLTAYLLLTVPAPSQAEGMDSVKEELQMRDMEPKLEDNGALNSNREGPALTRFSSSNRRNQRSIGLGSKYFPVTRSKYPIELELLVTNEEAERPKRFDDYGHMRFGKRGGDDQFDDYGHMRFGR

>Dwil SK dwil_GLEANR_11269

MSHRRCNICALALPLFVFIFYFLMVVPTPCKSASLEVGKQEQQLQDLETKMDTGAGPSDGDTFGISTGRFENRRNQRSIGFGPKSMKISRSRIPIELDFLLDNDDERLKSKRFDDYGHMRFGKRGGGEDQFDDYGHMRFGR

>Dyak SK dyak_GLEANR_14794

MGLRSCTHFATLVIPLWALAFCFLVVVPVPAQTNLQTSKGDRRLQDLESNMGAESDQPNANLVRPSLSRFGDKRNQKIITFGRRVPRPMIPIELDLLMDNDDENTKAKRFDDYGHMRFGKRGGDDQFDDYGHMRFGR

**short Neuropeptide F**

>Dana sNPF dana_GLEANR_15206

MFNLKPQLSQGWALALICLVCLQMQQPADAELPPVQGDKDTQSSVGQPIIKDSFAGASLNNLYDNLLQREYAGPVVFPNHQVERKAQRSPSLRLRFGRSDPDMLNNIVEKRWFGDVNQKPIRSPSLRLRFGRRDPNLPQMRRTAYDDLLERELTLNNQQPQLQEQLGSAGDSDLDADYDDLYKRVVRKPQRLRWGRSVPQFEDNDRDDFRKQIVAEKIHNLLMALQQYESPSARPNEDEVDLEEDSSEFQREARKPMRLRWGRSTGKGPSDQK

>Dere sNPF dere_GLEANR_5947

MFNLKRELSQGCALALICLVTLQMQQPTQAEVSSAQGDHPVQPPPEKQSSKDSFVGTPLSNLYDNLLQREYAGPVVFPNHQVERKAQRSPSLRLRFGRSDPDMLNSIVEKRWFGDVNQKPIRSPSLRLRFGRRDPSLPQMRRTAYDDLLERELTLNSQQQQQLGSEADSDLGADYDGLYERVVRKPQRLRWGRSVPQFEANNADNEQVERSQWYNSLLNSDKMRRMLVALQQQYEMPENLASYANEEDTDADLNSDTSEFQREVRKPMRLRWGRSTGKAPSEQK

>Dgri sNPF dgri_GLEANR_13551

MMQSKPQKIFGCALVLFSINLLMIQQSSAELSTVQGAPINNLYDNLLQREYAGPIVFPNHQVERKAQRSPSLRLRFGRSDPDMLNNIVEKRWFGDVNQKPIRSPSLRLRFGRRSDPNLPQMRRTAYDDLLERELTLNSQQVNKPATGNDAVVSDDDDAAFERMVRNPQRLRWLRSIMTNILDQDKSERHQLERDQLEREWLNVQELSRMLLAQKQQYEKSAAERAAIDAFYAIDSDEDQDENNSEYQFQREARKPMRLRWGRSTGKAPAEQKMPLAAETASVAPKSQN

>Dmel sNPF CG13968 spliceB

MFHLKRELSQGCALALICLVSLQMQQPAQAEVSSAQGTPLSNLYDNLLQREYAGPVVFPNHQVERKAQRSPSLRLRFGRSDPDMLNSIVEKRWFGDVNQKPIRSPSLRLRFGRRDPSLPQMRRTAYDDLPERELTLNSQQQQQLGSEPNFDLGADYDGLYERVVRKPQRLRWGRSVPQFESNNADNEQIERSQWYNSLLNSDKMRRMLVALQQQYEIPENVASYANDEDTDADLNNDTSEFQREVRKPMRLRWGRSTGKAPSEQKHTPEETSSIPPKTQN

>Dmel sNPF CG13968 spliceA

MFHLKRELSQGCALALICLVSLQMQQPAQAEVSSAQGEHLVQPPPEKQSSKDSFLGTPLSNLYDNLLQREYAGPVVFPNHQVERKAQRSPSLRLRFGRSDPDMLNSIVEKRWFGDVNQKPIRSPSLRLRFGRRDPSLPQMRRTAYDDLLERELTLNSQQQQQQLGTEPDSDLGADYDGLYERVVRKPQRLRWGRSVPQFEANNADNEQIERSQWYNSLLNSDKMRRMLVALQQQYEIPENVASYANDEDTDTDLNNDTSEFQREVRKPMRLRWGRSTGKAPSEQKHTPEETSSIPPKTQN

>Dmoj sNPF dmoj_GLEANR_1358

MMHLKSQLSYGCALALFSLNLFMLSSAELASAQGAAISNLYDNLLQREYAGPVVFPNHQVERKAQRSPSLRLRFGRSDPDMLNNIVEKRWFGDVNQKPIRSPSMRLRFGRRSDPNLPQMRRTAYDELLERELTLNNQQQLLNQPAAGYDADLSEDYDAGFERAVRKPQRLRWGRSVITGILNLGNNRLDKEQRIQAEIEQYLRQAQEVTRMLKALQQEYDIPAVERALESNEDQDDEQGEENAEYQFQRESRKPMRLRWGRSTGKAPAEQKQMPLAAAVSAETASVAPKLQN

>Dper sNPF dper_GLEANR_8872

MFRFNPQLSHGCALALICCLLNLLMMHQPTNAELSPVVQGGGPISNLYDNLLQREYAGPVVFPNHQVERKAQRSPSLRLRFGRSDPDMLNNIVEKRWFGDVNQKPIRSPSLRLRFGRRDPTLPQMRRTAYDDLLERELTLNNQQQQQLGDTADDLSADYDGLYERVVRKPQRLRWGRSVPQFEATNADNDQLYNSLWNSEKMRRMLLALQQYEAAPGHVAGYANDGDDTEAQLDEDTSEFQREARKPMRLRWGRSTGKAPATEQKIFMAKAKGTPKDNGKDIK

>Dpse sNPF dpse_GLEANR_9035

MFRFNPQLSHGCALALICCLLNLLMMHQPTNAELSPVVQGGGPISNLYDNLLQREYAGPVVFPNHQVERKAQRSPSLRLRFGRSDPDMLNNIVEKRWFGDVNQKPIRSPSLRLRFGRRDPTLPQMRRTAYDDLLERELTLNNQQQQQLGDTADDLSADYDGLYERVVRKPQRLRWGRSVPQFEATIGDNDQLYNSLWNSEKMRRMLLALQQYEAAPGHVAGYANDGDDTEAQLDEDTSEFQREARKPMRLRWGRSTGKAPATEQKIFMAKAKGTPKDNGKDIK

>Dsec sNPF dsec_GLEANR_18725

MFHLKRELSQGCALALICLVSLQMQQPAQAEVSSAQGEHPVQPPPEKQSSKDSFLGTPLSNLYDNLLQREYAGPVVFPNHQVERKAQRSPSLRLRFGRSDPDMLNSIVEKRWFGDVNQKPIRSPSLRLRFGRRDPSLPQMRRTAYDDLLERELTLNSQQQQQLGSEPDSDLGADYDGLYERVVRKPQRLRWGRSVPQFEANNADNEQIERSQWYNSLLNSDKMRRMLVALQQQYEIPENVASYANEEDTDAELNNDTSEFQREVRKPMRLRWGRSTGKAPSEQKHTPEETSSIPPKTQN

>Dsim sNPF dsim_GLEANR_7961

MFHLKRELSQGCALALICLVSLQMQQSAQAEVSSAQGEHPVQSPPEKQSSKDSFLGTPLSNLYDNLLQREYAGPVVFPNHQVERKAQRSPSLRLRFGRSDPDMLNSIVEKRWFGDVNQKPIRSPSLRLRFGRRDPSLPQMRRTAYDDLLERELTLNSQQQQQLGSEPDSDLGADYDGLYERVVRKPQRLRWGRSVPQFEANNADNEQIERSQWYNSLLNSDKMRRMLVALQQQYEIPENVASYANEEDTDAELTNDTSEFQREVRKPMRLRWGRSTGKAPSEQKHTPEETSSIPPKTQN

>Dvir sNPF dvir_GLEANR_2893

MMYFKSQLSYGCALALFSLNLLMLQQSSAELSTVQGAPISSLYDNLLQREYAGPIVFPNHQVERKAQRSPSLRLRFGRSDPDMLNNIVEKRWFGDVNQKPIRSPSLRLRFGRRSDPNLPQMRRTAYDDLLERELTLNNQQANQPGAGYDAELSDDYDVAFERAVRKPQRLRWGRSVMSSPLDLGNNRVDKDQQRERTQLEREWLKWQEMTRMLLALQQQYDSSAGELPGGSGEDTDEDQDEDNSEYQFQREARKPMRLRWGRSTGKAPVEQKKMPLAAETASVAPKSEN

>Dwil sNPF dwil_GLEANR_19356

MFHFKIQQLYVGYALAFFSLTLMMLQPSSAELKTNAAPISNLYDNLLQREYAGPVVFPNHQVERKAQRSPSLRLRFGRSDPDMLNNIVEKRWFGDVNQKPIRSPSLRLRFGRRSDPNLPQMRRTAYDDLLERELTLNSQQQQPAAFDDAVDYDSLYERVVRKPQRLRWGRSVPQYEAPQLDNEQLERNELYNSLLSSEKMRRMLMALQQYESAPADLSDTEEVEDDQDTSEFQREARKPMRLRWGRSTGKAPTDQGKHTAMGASDENSPTASAAKIHN

>Dyak sNPF dyak_GLEANR_13516

MFHLKRELSQGCALALICLVTIQMQQPTQAEASSAQGDHHPVHPTPKKQYSKDFFAGTPLSNLYDNLLQREYAGPVVFPNHQVERKAQRSPSLRLRFGRSDPDMLNSIVEKRWFGDVNQKPIRSPSLRLRFGRRDPNLPQMRRTAYDDLLERELTLNGQQQQLASESDSDLGADYDGLYERVVRKPQRLRWGRSVPQFEANNADNEQIERSQWYNSLLNSDKMRRMLVALQQQYEMPENVASYANDEDTDAELNNDASEFQREVRKPMRLRWGRSTGKAPSEQKPTPEETSSISPKTQN

**Drosophila tachykinins (Dtks)**

>Dana TK dana_GLEANR_19016

MRSQSGTIAVAVLLLLLLTAASTAAEADLAGESPGVSTLPPSVEQPRRVVKRAPTSSFIGMRGKKDDERDTESSDISEGNWLGNGPDPLDYADLESYYSENGRRLKKAPLAFVGMRGKKFIPMSVRLSGVLQHMEEERLREGLLQDFLNSLTGEENGAGDVDKRAPTGFNGMRGKRPALNGDEGEEEEAMELLQKRAPVNSFVGMRGKKDVSHQHYKRAALSEFWHTFFKKAYDVRGKKQRFADFNNKFVAVRGKKSEQDGEGAGNWEAIGQQQYLVHPWLNVWGDKRAPNGFLGMRGKRPALFE

>Dere TK dere_GLEANR_2080

MRPLSGLVAVALLLLLLLTASSSAADTGTEAEVSGSPLTPGPDESRRVVKRAPTSSFIGMRGKKDEERDTSEGNWLGSGPDPLDYADMEADSGYSENGRRLKKAPLAFMGMRGKKVFPINPRLYEVLQSLEEERLRESLLQDYFDRIAGYDGSAVGKRAPTGFTGMRGKRPALLAGEDDAEADEATELEQKRAPINSFVGMRGKKDVSHQHYKRAALSDSNDLRGKQQRFADFNSKFVAVRGKKSDLEGNGIGIGIGEDHEQSLVHPWLYLWGEKRAPNGFLGMRGKRP

>Dgri TK dgri_GLEANR_124*

MLLLLLLLTTVAVASGDIDGVAEDGDVHVDGISTLAPGTVTQPRKIVKRAPTNSFIGMRGKKELESSTDENWLGPDPLDYEGLVDVDNYYNENGRRLKKAPLAFVGMRGKKFNPNSNNNLLDLLQHMEEERVREYILQDFLDHLAIDGNGMAKRAPTGFTGMRGKRPTMTDGDAAAQDEDDAMELLQKRAPVNSFVGVRGKKDVSHQNYKRAALSEAYDVRGKKQRYADFNSKFVAVRGKKSELDDATGLEQNILQQQQPWVYVIGGKRAPNGFVGMRGKRPELVE

>Dmel TK CG14734-RA

MRPLSGLIALALLLLLLLTAPSSAADTETESSGSPLTPGAEEPRRVVKRAPTSSFIGMRGKKDEEHDTSEGNWLGSGPDPLDYADEEADSSYAENGRRLKKAPLAFVGLRGKKFIPINNRLSDVLQSLEEERLRDSLLQDFFDRVAGRDGSAVGKRAPTGFTGMRGKRPALLAGGDDAEADEATELQQKRAPVNSFVGMRGKKDVSHQHYKRAALSDSYDLRGKQQRFADFNSKFVAVRGKKSDLEGNGVGIGDNHEQALVHPWLYLWGEKRAPNGFLGMRGKRPALSE

>Dmoj TK dmoj_GLEANR_9239

MCNKKLLLLLLLLTVAAVASGAEAEPEDADAMTISPATPGAQEEARNMVKRAPTSSFIGMRGKKEQDASADANWFGPDPLDYGEEDEDNSYYENGRRLKKAPMAFVGMRGKKFSPNTNRLRDLLQNMEEQRLRESFLQEFLNNLATDGGDVAKRAPTGFTGMRGKRPSLSENEDDEDDALELLEKRAPVNSFVGMRGKKDVSHQHYKRAALSELWRQLSKKAFDVRGKKQRYADFNSKFVAVRGKKSALSDANGAAEENLIQPWVYVIGGKRAPSGFLGMRGKRPALVE

>Dper TK dper_GLEANR_3683

MRSQGGSFAVALLLLLLLTAASTAADAEPDVESSASTLPPGADAPRRMVKRAPTSSFIGMRGKKEDEKDQRAADWMGPDPLDYADMDEDSIYYENGKRLKKAPMSFVGMRGKKYIPISNRLSDVLHQIEEQRMRENLLEDLFERLAAGDDSVGDVGKRAPTGFTGMRGKRPMSGDDDDNDAMELLQKRAPVNSFLGVRGKKDVSHQHYKRAALSEFWHNFLKKAYDVRGKKERYADFNSKFVAVRGKKSEQDAGLDTGDGVGDGDQQYLVRPWLYLWADNKRAPSGFQGMRGKRPGPILNP

>Dpse TK >dpse_GLEANR_3332

MRSQGGSFAVALLLLLLLTAAATAADAEPDVESSVSTLPPGADAPRRMVKRAPTSSFIGMRGKKEDEKDQRAADWMGPDPLDYADMDEDSIYYENGKRLKKAPMSFVGMRGKKYIPISNRLSDVLHQIEEQRMRENLLEDLFERLAAGDDSVGDVGKRAPTGFTGMRGKRPMSGDDDDNDAMELLQKRAPVNSFLGVRGKKDVSHQHYKRAALSEFWHNFLKKAYDVRGKKERYADFNSKFVAVRGKKSEQEAGLDTGDGDGDQQYLVRPWLYLWADNKRAPSGFQGMRGKRPALAE

>Dsec TK dsec_GLEANR_8966

MRPLSGLIAVALLLLLLLTAPSSAADTETESSGSPLTPGAEEPRRVVKRAPTSSFIGMRGKKDEEHDTSEGNWLGSGPDPLDYADEETDNSYSENGRRLKKAPLAFVGLRGKKFIPINTRLSDVLQSLEEERLRDSLLQDFFDRVAGRDGSAVGKRAPTGFTGMRGKRPALLAGDDDAEADEAMELQQKRAPVNSFVGMRGKKDVSHQHYKRAALSDSYDLRGKQQRFADFNSKFVAVRGKKSDLEGNGVGIGEDHEQALVHPWLYLWGEKRAPNGFLGMRGKRPALFE

>Dsim TK dsim_GLEANR_4386

MRPLSGLIAVALLLLLLLTAPSSAADTETESSGSPLTPGAEEPRRVVKRAPTSSFIGMRGKKDEEHDTSEGNWLGSGPDPLDYADEEADSSYSENGRRLKKAPLAFVGLRGKKFIPINTRLSDVLQSLEEERLRDSLLQDFFDRVAGRDGSVVGKRAPTGFTGMRGKRPALLAGDDDAEADEAMELQQKRAPVNSFVGMRGKKDVSHQHYKRAALSDSYDLRGKQQRFADFNSKFVAVRGKKSDLEGNGVGIGEDHEQALVHPWLYLWGEKRAPNGFLGMRGKRPALFE

>Dvir TK dvir_GLEANR_9020

MLNQQLLVVLLLTAAVVASSDVEAEAEDVDASTFSTSAPGAEPQPRNMVKRAPNLSFIGMRGKKEQEPSADDNWLGPDPLDYTEDDAGNFYNENMRRLKKAPLAFVGLRGKKFTPNNNRLHDLLQQMEEDRLRENLLQDFLEHLATDGNDVAKRAPTGFTGMRGKRPSIAEYDAVENGEDAMELLAKRAPVNSFVGVRGKKDVSHQHYKRAALSELWRKIFKKAYDVRGKKQRYADFNSKFVAVRGKKSNVEDEAGLEPNAMRPWVYLIGGKRAPSGFLGMRGKRPALAE

>Dwil TK dwil_GLEANR_11855

MANAAISEGDENNLSSTATSLQQEPRKLVKRAPAAGFIGMRGKKELETDTETDRAYPVSNDNWLGPDPLDYGEDSDDYYENGRRLKKAPTAFVGMRGKKYTPSSNRLSNLLRQIEEQRLRENVLQELFDRLADQNSVGDAEIPNKRAPTGFTGMRGKRPAEDDDDAMELFEKRAPINAFVGVRGKKDVSHQNYKRAAPLSEAYDARGKKHRFVDFNNKFVAVRGKKNNLDLEDGQDQYLVHPWSYLLNEKRAPNGFVGMRGKRPVLALE

>Dyak TK dyak_GLEANR_8245

MRPLSGLLAVTLLLLLLLTAPSSAADTETEVSGSPLTPGADETRRVVKRAPTSSFIGMRGKKDEERDTSEGNWLGNSGPDPLDYADEEADSSYSENGRRLKKAPLAFVGMRGKKFIPINTRLSEVLQSLEEERLRESLLQDYFDRLGSAVGKRAPTGFTGMRGKRPALLAGDDDADADEATELHEKRAPVNSFVGMRGKKDVSHQHYKRAALSDSYDLRGKQQRFADFNSKFVAVRGKKSDLEGNGIGTGIGEDHEQSLVHPWLYLWGEKRAPNGFLGMRGKRP

*A second sequence dgri_GLEANR_1183 was found at scaffold_14701:4144..2888 (- strand)which is nearly identical to dgri_GLEANR_124 at scaffold_15074:6115935..6115288 (- strand)

LLQHMEEERVREYILQDFLDHLAIDGHGMAKRAPTGFTGMRGKRPTMTEGDAAAQDEDDAMELLQKRAPVNSFVGVRGKKDVSHQNYKRAALSEAYDVRGKKQRYADFNSKFVAVRGKKSELDDATGLEQNILQQQQPWVYIIGGKRAPNGFVGMRGKRPGEECRCSWTQLDHRPCLLYAYQCTIAELVE
