# Supplementary material for: Molecular evolution of neuropeptides in the genus Drosophila
Source: Genome Biol. 2008 Aug 21;9(8):R131. doi: 10.1186/gb-2008-9-8-r131 (PMC2575521; doi:10.1186/gb-2008-9-8-r131)
Supplement: Additional data file 5 — Overall average amino acid distances Dso. [file gb-2008-9-8-r131-S5.doc]

**Table S1**

| Peptid | distance | s.d. | copy number |
| --- | --- | --- | --- |
| AKH | 0 | 0 | 1 |
| ASTc | 0 | 0 | 1 |
| CAPA-PK | 0.024 | 0.017 | 1 |
| CCAP | 0 | 0 | 1 |
| corazonin | 0 | 0 | 1 |
| DH31 | 0.018 | 0.019 | 1 |
| DH44 | 0.012 | 0.012 | 1 |
| Drosokinin | 0 | 0 | 1 |
| HUG-PK | 0 | 0 | 1 |
| HUG-gamma | 0.259 | 0.124 |  |
| IFa | 0 | 0 | 1 |
| MS | 0 | 0 | 1 |
| NPF | 0.134 | 0.045 | 1 |
| NPLP1_ASP | 0.056 | 0.037 | 1 |
| NPLP1_MTY | 0.093 | 0.048 | 1 |
| NPLP1_IPN | 0.024 | 0.018 | 1 |
| PDF | 0.041 | 0.03 | 1 |
| Proctolin | 0 | 0 | 1 |
| CAPA-PVK1 | 0.042 | 0.031 | 2 |
| CAPA-PVK2 | 0.205 | 0.099 | 2 |
| ETH1 | 0.025 | 0.024 | 2 |
| ETH2 | 0.267 | 0.135 | 2 |
| SK0 | 0.601 | 0.201 | 3 |
| SK1 | 0 | 0 | 3 |
| SK2 | 0.056 | 0.029 | 3 |
| ASTa1 | 0.06 | 0.065 | 4 |
| ASTa2 | 0.065 | 0.041 | 4 |
| ASTa3 | 0 | 0 | 4 |
| ASTa4 | 0 | 0 | 4 |
| sNPF1 | 0 | 0 | 4 |
| sNPF2 | 0.326 | nd | 4 |
| sNPF3 | 0 | 0 | 4 |
| sNPF4 | 0 | 0 | 4 |
| MIP1 | 0.036 | 0.04 | 5 |
| MIP2 | 0.052 | 0.049 | 5 |
| MIP3 | 0 | 0 | 5 |
| MIP4 | 0 | 0 | 5 |
| MIP5 | 0.05 | 0.055 | 5 |
| DTK1 | 0.102 | 0.07 | 6 |
| DTK2 | 0.187 | 0.097 | 6 |
| DTK3 | 0.02 | 0.021 | 6 |
| DTK4 | 0.135 | 0.073 | 6 |
| DTK5 | 0.135 | 0.063 | 6 |
| DTK6 | 0.13 | 0.108 | 6 |
| FMRF1 | 0.127 |  | 10 |
| FMRF2-1 | 0.093 |  | 10 |
| FMRF2-2 | 0.179 |  | 10 |
| FMRF2-3 | nd |  | 10 |
| FMRF2-4 | 0 |  | 10 |
| FMRF2-5 | 0.17 |  | 10 |
| FMRF2-6 | 0.167 |  | 10 |
| FMRF3 | 0.172 |  | 10 |
| FMRF2-7 | nd |  | 10 |
| FMRF2-8 | nd |  | 10 |
| FMRF2-9 | nd |  | 10 |
| FMRF2-10 | nd |  | 10 |
| FMRF2-11 | nd |  | 10 |
| FMRF3-2 | 0.208 |  | 10 |
| FMRF4 | 0.11 |  | 10 |
| FMRF5 | 0.04 |  | 10 |
| FMRF6 | 0 |  | 10 |
| FMRF7 | 0.748 |  | 10 |
| FMRF8 | 0.064 |  | 10 |
